# Methodological Approach to Identify and Expand the Volume of Antimicrobial Resistance (AMR) Data in the Human Health Sector in Low- and Middle-Income Countries in Asia: Implications for Local and Regional AMR Surveillance Systems Strengthening

**DOI:** 10.1093/cid/ciad634

**Published:** 2023-12-20

**Authors:** Hea Sun Joh, Corin Yeats, Alina Shaw, Nimesh Poudyal, Patrick Gallagher, Jong-Hoon Kim, Affan Shaikh, Hye Jin Seo, Kyu-young Kevin Chi, Kristi Prifti, Alyssa Cho, Mohammad Julhas Sujan, Emmanuel Eraly, Kien Duc Pham, Subha Shrestha, Ahmed Taha Aboushady, Gideok Pak, GeunHyeog Jang, Eun Lyeong Park, Hyeong-Won Seo, Khalil Abudahab, Ben E W Taylor, Adam Clark, Brooke Dolabella, Hyein Yoon, Jihyun Han, Soo Young Kwon, Florian Marks, John Stelling, David M Aanensen, William R MacWright, Marianne Holm

**Affiliations:** International Vaccine Institute, Seoul, Republic of Korea; Centre for Genomic Pathogen Surveillance, Big Data Institute, University of Oxford, Oxford, United Kingdom; Public Health Surveillance Group, LLC, Princeton, New Jersey, USA; International Vaccine Institute, Seoul, Republic of Korea; Public Health Surveillance Group, LLC, Princeton, New Jersey, USA; International Vaccine Institute, Seoul, Republic of Korea; Public Health Surveillance Group, LLC, Princeton, New Jersey, USA; International Vaccine Institute, Seoul, Republic of Korea; International Vaccine Institute, Seoul, Republic of Korea; International Vaccine Institute, Seoul, Republic of Korea; International Vaccine Institute, Seoul, Republic of Korea; International Vaccine Institute, Seoul, Republic of Korea; International Vaccine Institute, Seoul, Republic of Korea; International Vaccine Institute, Seoul, Republic of Korea; International Vaccine Institute, Seoul, Republic of Korea; International Vaccine Institute, Seoul, Republic of Korea; Brigham & Women's Hospital, Harvard Medical School, Boston, Massachusetts, USA; International Vaccine Institute, Seoul, Republic of Korea; International Vaccine Institute, Seoul, Republic of Korea; International Vaccine Institute, Seoul, Republic of Korea; International Vaccine Institute, Seoul, Republic of Korea; Centre for Genomic Pathogen Surveillance, Big Data Institute, University of Oxford, Oxford, United Kingdom; Centre for Genomic Pathogen Surveillance, Big Data Institute, University of Oxford, Oxford, United Kingdom; Brigham & Women's Hospital, Harvard Medical School, Boston, Massachusetts, USA; Public Health Surveillance Group, LLC, Princeton, New Jersey, USA; International Vaccine Institute, Seoul, Republic of Korea; International Vaccine Institute, Seoul, Republic of Korea; International Vaccine Institute, Seoul, Republic of Korea; International Vaccine Institute, Seoul, Republic of Korea; Cambridge Institute of Therapeutic Immunology and Infectious Disease, University of Cambridge School of Clinical Medicine, Cambridge, United Kingdom; Heidelberg Institute of Global Health, University of Heidelberg, Heidelberg, Germany; Madagascar Institute for Vaccine Research, University of Antananarivo, Antananarivo, Madagascar; Brigham & Women's Hospital, Harvard Medical School, Boston, Massachusetts, USA; Centre for Genomic Pathogen Surveillance, Big Data Institute, University of Oxford, Oxford, United Kingdom; Public Health Surveillance Group, LLC, Princeton, New Jersey, USA; International Vaccine Institute, Seoul, Republic of Korea

**Keywords:** CAPTURA, Antimicrobial Resistance (AMR), project implementation, AMR surveillance, capacity building

## Abstract

Antimicrobial resistance (AMR) is a multifaceted global health problem disproportionately affecting low- and middle-income countries (LMICs). The Capturing data on Antimicrobial resistance Patterns and Trends in Use in Regions of Asia (CAPTURA) project was tasked to expand the volume of AMR and antimicrobial use data in Asia. The CAPTURA project used 2 data-collection streams: facility data and project metadata. Project metadata constituted information collected to map out data sources and assess data quality, while facility data referred to the retrospective data collected from healthcare facilities. A down-selection process, labelled “the funnel approach” by the project, was adopted to use the project metadata in prioritizing and selecting laboratories for retrospective AMR data collection. Moreover, the metadata served as a guide for understanding the AMR data once they were collected. The findings from CAPTURA's metadata add to the current discourse on the limitation of AMR data in LMICs. There is generally a low volume of AMR data generated as there is a lack of microbiology laboratories with sufficient antimicrobial susceptibility testing capacity. Many laboratories in Asia are still capturing data on paper, resulting in scattered or unused data not readily accessible or shareable for analyses. There is also a lack of clinical and epidemiological data captured, impeding interpretation and in-depth understanding of the AMR data. CAPTURA's experience in Asia suggests that there is a wide spectrum of capacity and capability of microbiology laboratories within a country and region. As local AMR surveillance is a crucial instrument to inform context-specific measures to combat AMR, it is important to understand and assess current capacity-building needs while implementing activities to enhance surveillance systems.

## Teaser

We present the project's methodological approach to identify, select, and grade laboratories for antimicrobial resistance (AMR) data collation and analyses. The findings of the project’s metadata, information collected to map out data sources and assess data quality, indicate current capacity-building needs in microbiology laboratories in low- and middle-income countries.

Antimicrobial resistance (AMR) is a multifaceted global health problem that disproportionately affects low- and middle-income countries (LMICs), with the highest burden and heaviest consequences predicted in the most impoverished populations [1]. Local AMR surveillance is a crucial instrument to inform appropriate, context-specific measures to combat AMR. However, in low-resource settings, limited data availability and issues with data quality are a significant hindrance for effective surveillance. Therefore, increasing AMR data and/or the capacity to improve data quality and the use of data in LMICs is needed [2].

Since the endorsement of the Global Action Plan on Antimicrobial Resistance in 2015 and the Review on Antimicrobial Resistance published in 2016, several global initiatives have been established to enhance knowledge and evidence on AMR [2, 3], including the Fleming Fund (FF), which supports LMICs in generating, sharing, and using data for surveillance [4]. Four large regional FF grants initiated in early 2019 were tasked to “expand the volume of historical and current data on AMR and antimicrobial usage” [4].

**Figure 1.**
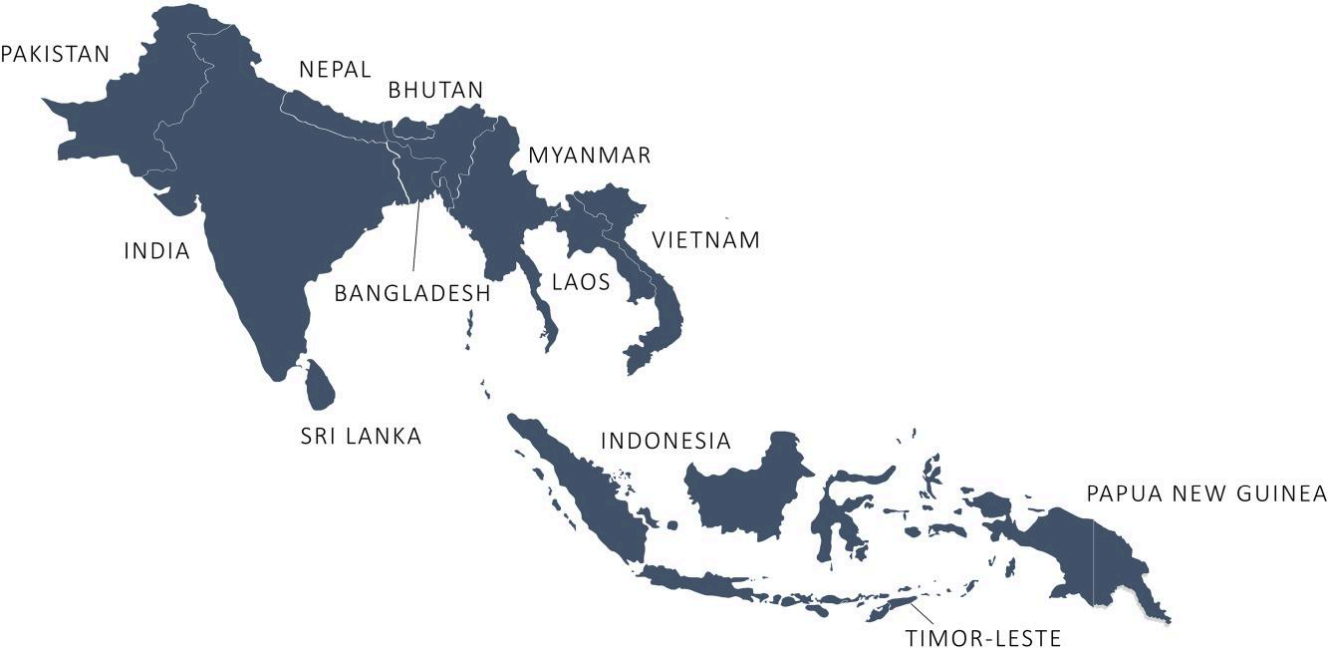
Map of 12 CAPTURA countries in South and Southeast Asia. Abbreviation: CAPTURA, Capturing data on Antimicrobial resistance Patterns and Trends in Use in Regions of Asia.

The Capturing data on Antimicrobial resistance Patterns and Trends in Use in Regions of Asia (CAPTURA) Consortium, led by the International Vaccine Institute (IVI) in partnership with the Public Health Surveillance Group, WHONET development team at the Brigham and Women's Hospital, and Big Data Institute at the University of Oxford, was granted 2 of the 4 regional grants to expand the volume of historical and current AMR and antimicrobial use (AMU) data in 12 countries across South Asia and Southeast Asia [5]. The countries were preselected by the FF and included the following: Bangladesh, Bhutan, India, Indonesia, Laos, Myanmar, Nepal, Pakistan, Papua New Guinea, Sri Lanka, Timor-Leste, and Vietnam (Figure 1). Since local contexts and levels of AMR efforts are highly variable across countries [6], this required a standardized and thorough, but also adaptive and flexible, approach to initial data source identification and prioritization. To ensure successful implementation of the project, the scope of work in each country was tailored to consider feasibility and needs expressed by stakeholders while also applying standardized and coherent methods to identify and assess relevant data sources across the regions [5]. This paper illustrates these processes and summary findings of project metadata collected as part of the identification, selection, and grading of laboratories to enable retrospective AMR data collation and analyses by the CAPTURA project in the South Asia and Southeast Asian regions.

## METHODS

**Figure 2.**
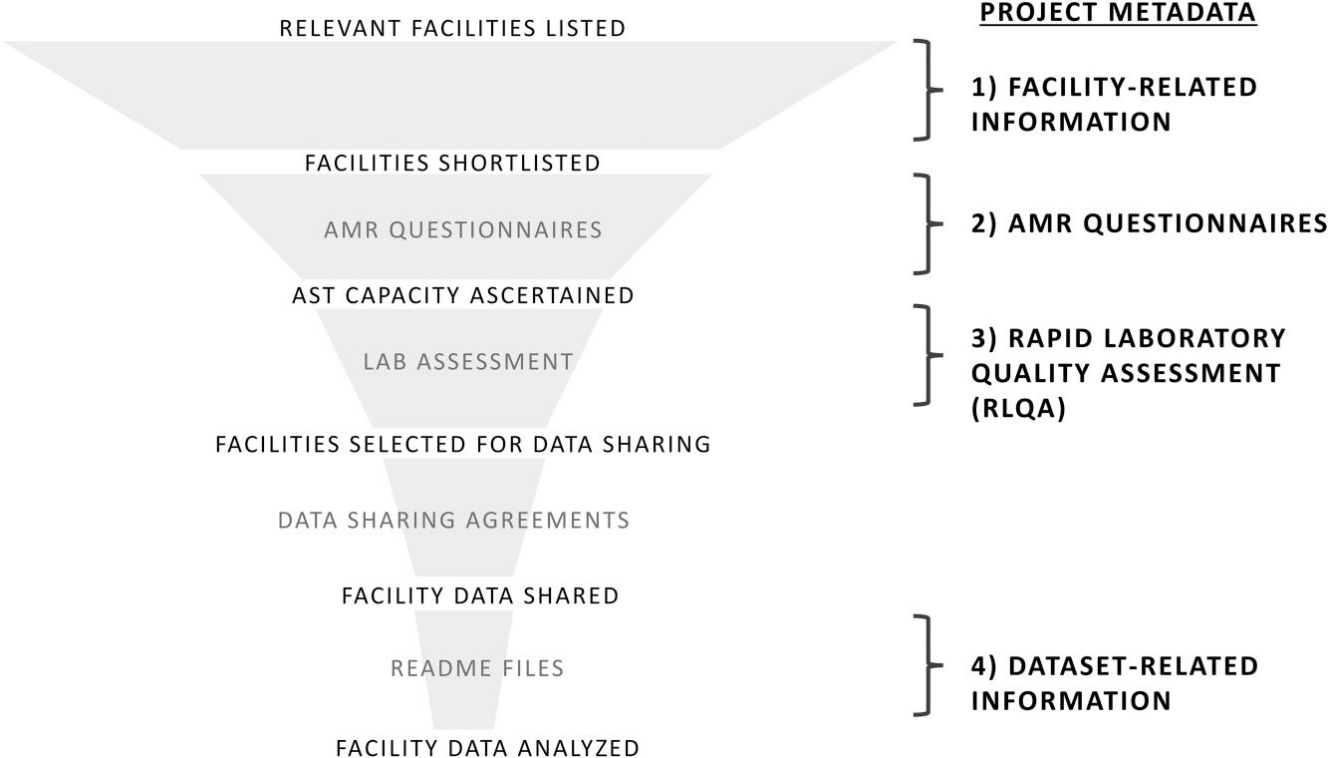
CAPTURA's funnel approach for site selection, data collection, and analyses using project metadata. Abbreviations: AMR, antimicrobial resistance; CAPTURA, Capturing data on Antimicrobial resistance Patterns and Trends in Use in Regions of Asia.

The CAPTURA project used 2 distinct data-collection streams—namely, facility data and project metadata. Facility data refer to the retrospective data (antimicrobial resistance, use, and consumption) collected directly from healthcare facility records, whereas project metadata constituted all the information collected to map out data sources and assess data quality. In detail, the project metadata consisted of (1) facility-related information, (2) AMR and AMU questionnaires, (3) Rapid Laboratory Quality Assessment (RLQA), and (4) other dataset-related information. A down-selection funnel approach using the information from the project metadata was adopted for retrospective AMR data collection (Figure 2).

Facility-related information provided names and locations of healthcare facilities. These facilities were mostly acute care hospitals, ranging from primary to tertiary hospitals. In addition, national reference laboratories focusing on reference testing and disease surveillance were included. This information was gathered from landscape and desktop reviews and interviews with key stakeholders during virtual and in-person meetings with local AMR Coordinating Committees (AMR CC). The variables included geocoordinates of the facilities (latitude and longitude), affiliation (private/public/non-governmental organization/military), and possible types of data present. The master list generated with this basic information served as a starting point to plan the project in each country and was also shared with relevant local AMR stakeholders to receive their input to shortlist facilities for inclusion in the project. The master list also served as a directory for the CAPTURA team to indicate status of engagement and to keep track of collected data.

The AMR and AMU questionnaires captured basic information about the healthcare facility, including the presence and format of AMR/AMU data and practices of data capture and storage. The AMR questionnaire (also called the laboratory questionnaire) was conducted in laboratories, while the AMU questionnaire (also called the pharmacy questionnaire) was conducted in pharmacies within the healthcare facilities listed the master list. Some private pharmacy shops/stalls were surveyed as not all hospitals had pharmacies on the premises.

Each questionnaire was filled out by the respondent (laboratory staff for AMR questionnaire and pharmacist for AMU questionnaire) and took approximately 10–15 minutes to complete. Translated questionnaires, prepared by bilingual country coordinators hired by the project and based in-country, were developed for countries where the local language was preferred. The questionnaires were deployed in-person or online. SurveyMethods, an existing online survey tool, was adopted for online deployment of the questionnaires [7]. Recognizing that not all facilities could be visited by the country team, especially during the coronavirus disease 2019 (COVID-19) pandemic, online versions were circulated via URL sharing. This method was particularly useful for reaching out to locations in remote and/or rural areas. Online responses were exported into comma-separated values (CSV) files, and any incomplete entries or double entries were checked for exclusion. The responses to the questionnaires were stored in EpiCollect, a free versatile software that can be used to build structured surveys, deploy them into the field using mobile devices, and load data into a centralized database for analysis [8]. Country coordinators were trained by the consortium on the use of the software, and if required, they provided further training to data collectors in the country (eg, Bangladesh and Nepal). Country-specific EpiCollect projects were created enabling convenient use of the database by the country team (country coordinator and other staff based in-country) and country point of contact from the consortium. The AMR and AMU questionnaires and illustration on deployment methods can be found in Supplementary Appendices 1, 2, and 3.

For participation in the RLQA, the AST capacity of laboratories was first confirmed using the AMR questionnaire. In some instances, interviews with key stakeholders confirmed AST capacity, enabling a laboratory to be assessed without completing the AMR questionnaire. The RLQA was used to assess the quality of laboratories at present as well as within the last 3 years through recall by local laboratory staff (equivalent to the retrospective data-collection period). The RLQA was developed referencing existing laboratory assessment tools, such as the Global Antimicrobial Resistance and Use Surveillance System (GLASS) Laboratory Assessment Questionnaire for Antimicrobial Resistance Testing, Mott MacDonald Laboratory Assessment, and the Centers for Disease Control and Prevention/Global Health Security Agenda Lab Microbiology Assessment Tools [9, 10]. Feedback from country coordinator(s), World Health Organization (WHO) representatives, and other key stakeholders to the tool was also incorporated prior to finalizing. The tool was initially designed to be administered in-person, for approximately 1 to 1.5 hours, by a trained CAPTURA country microbiologist or coordinator. When the COVID-19 pandemic was declared, conducting RLQAs in-person became difficult due to lockdowns and travel restrictions. Therefore, in November 2020, virtual RLQA administration was introduced [11].

The RLQA is divided into 7 sections consisting of 126 questions in total. The sections are Equipment, Staffing, Media, Pathogen Identification, Antimicrobial Susceptibility Testing (AST), Internal Quality Control (IQC), and External Quality Assurance (EQA). Each section has questions specifically designed to address the retrospective quality of different areas of the laboratory, including human resources, equipment availability, status of supplies, and quality-control standards implemented at the laboratory over the last 3 years. The last part of the assessment requires the assessor to conduct a visual inspection of different units and practices at the laboratory to verify the responses. Each section has a different weight for the overall score (out of 100): Equipment (10%), Staffing (20%), Media (10%), Pathogen Identification (15%), AST (20%), IQC (15%), and EQA (10%); some questions in the sections have a higher number of points associated as they were deemed highly critical to quality. The responses of RLQAs were subject to automatic scoring using a JavaScript following a scoring scheme. A Data-flo, or web-based data manipulation and integration tool, was used to extract the results [12]. The scores were then used for prioritization of sites for CAPTURA to approach for data sharing and, if applicable, to inform prioritization of datasets to be included in the national- and/or regional-level analyses. The RLQA, including its scoring guide and illustration on deployment methods, can be found in Supplementary Appendices 4 and 5.

Dataset-related information was compiled in a Microsoft Word document in the form of a readme file, which had a series of questions relating to the dataset shared with the project. Questions, such as general description (time period and geographic relevance, presence of data dictionary), quality (criteria used to collect or exclude data, any changes in guidelines and protocols during data collection), and denominators (population data, number of admissions), were asked to data managers assisting in data export. The readme file templates can be found in Supplementary Appendices 6, 7 and 8. For datasets where data-related information was difficult to receive via the Word document, follow-up emails and data-review meetings were organized to gather necessary information. The aim of such collection was to acquire general information on data to include contextual insights during analyses and interpretation.

Sourced from CAPTURA's master list, the names and locations of the laboratories served as a basis in the mapping and visualization of the collected metadata. From the AMR/AMU questionnaire and RLQA, selected variables were prioritized and extracted from EpiCollect. The selected variables are listed in Supplementary Appendix 9. For each of the selected and extracted variables of project metadata, a “dataflow” was created within the Data-flo tool. These dataflows imported the variables from the EpiCollect projects and integrated them into a common representation, from which further dataflows were processed to generate national and regional aggregations. The outputs were saved as CSV files, which were then uploaded into the national and regional Microreact projects for further analyses and visualization. Microreact is a general-purpose analysis and visualization platform that links data tables with maps, charts, timelines, and network visualizations [13, 14]. For CAPTURA, Microreact dashboards displaying results of the metadata were created using the Vega-Lite Grammar, a specification of data visualization in a series of interactive charts [15]. The dashboards visualizing the metadata findings at facility, national, and regional levels were updated as new data became available or corrected during the project.

Although the AMU questionnaire was developed and deployed as part of the project metadata, it was not used to guide collection and analyses of retrospective AMU and antimicrobial consumption (AMC) data. Instead, comprehensive engagement and consultation with relevant in-country stakeholders were prioritized and conducted to identify AMU/AMC data sources and assess data quality [16]. The AMU questionnaire is mentioned in this paper to reflect the setup of the project metadata but excluded in the Results and Discussion sections.

The project was exempted for ethical review at the Institutional Review Board (IRB) of IVI since the project did not involve intervention or interaction with individuals and the information collected is not individually identifiable. This exemption is according to the definition of the IVI IRB SOP D-RB-4-003.

## RESULTS

The collection of project metadata was first initiated in 10 out of 12 CAPTURA countries. The 2 excluded countries were India and Myanmar, as project activities were limited to desktop review on current AMR surveillance networks. Among the countries with metadata collection, some were unsuccessful in compiling and incomplete in analyses because questionnaires were not shared and/or withheld for various reasons. In summary, a total of 152 AMR questionnaires were collected from 9 countries, and in 136 of these, AST capacity was confirmed. The total number of questionnaires collected in each country and the number of laboratories conducting AST are indicated in parentheses: Bangladesh (28/28), Bhutan (4/4), Indonesia (36/29), Laos (10/7), Nepal (41/39), Pakistan (16/16), Papua New Guinea (7/5), Sri Lanka (7/7), and Timor-Leste (3/1). In Sri Lanka, although the project collected the AMR questionnaire in both the public and private sectors, the results from the public sector were withheld by the government for use by the project.

**Figure 3.**
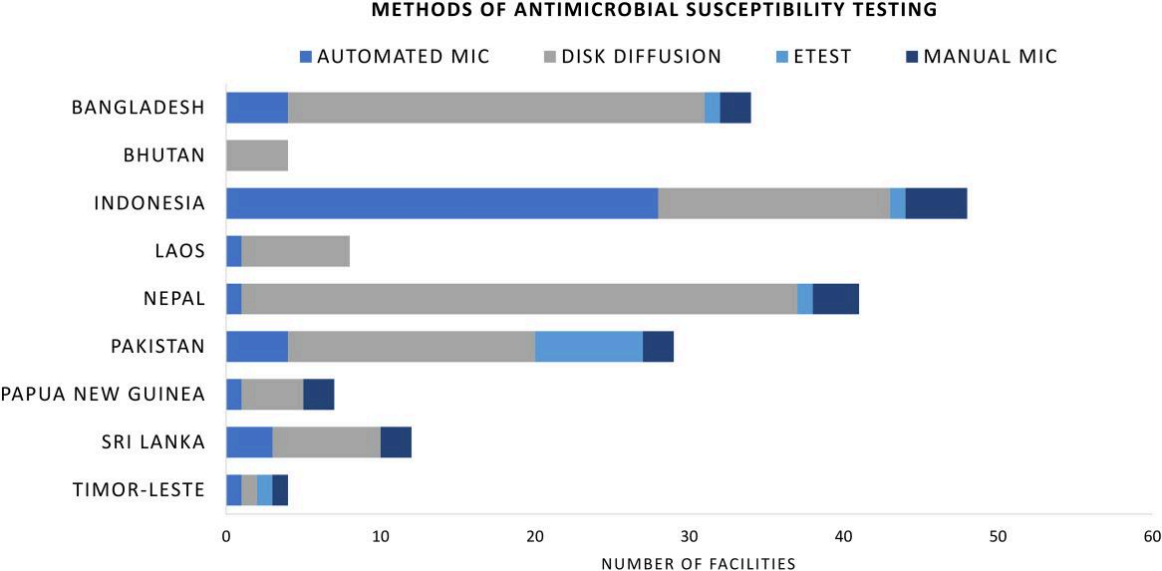
Common methods of antimicrobial susceptibility testing in CAPTURA countries. Abbreviations: CAPTURA, Capturing data on Antimicrobial resistance Patterns and Trends in Use in Regions of Asia; MIC, minimum inhibitory concentration.

Disk diffusion was the most common method of AST (86%, 117/136), especially in Bangladesh, Nepal, and Pakistan, followed by automated minimum inhibitory concentration (MIC)(32%, 43/136), which was most common in Indonesia (Figure 3). Forty percent (54/136) of the laboratories reported that they conducted fewer than 100 ASTs per month, while 44% (60/136) of the laboratories reported that they conducted between 101 and 1000 ASTs per month. Only 15% (21/136) of the laboratories reported conducting more than 1000 ASTs per month. Results from AST were commonly stored in both paper and electronic format (57%, 78/136), although there were laboratories where data were stored only in paper (24%, 33/136) or only in electronic (18%, 24/136) format. Of the AST data stored electronically, 49% of the laboratories (50/102) reported to have up to 3 years of data, while 37% (38/102) of the laboratories reported to have 3 to 10 years' worth of data. There were only a few laboratories with 10 years' worth of data (7%, 7/102). Of the laboratories conducting AST, 74% (101/136) reported conducting AMR analyses using their data, while 24% (33/136) reported not conducting any analyses. With regard to data sharing, asked as “isolate-level AMR data ever sent to another organisation or facility,” 47% (64/136) of the laboratories reported sharing data. The same proportion (47%, 64/136) reported not sharing any data outside of their laboratory. Reasons for not sharing data were not asked.

**Figure 4.**
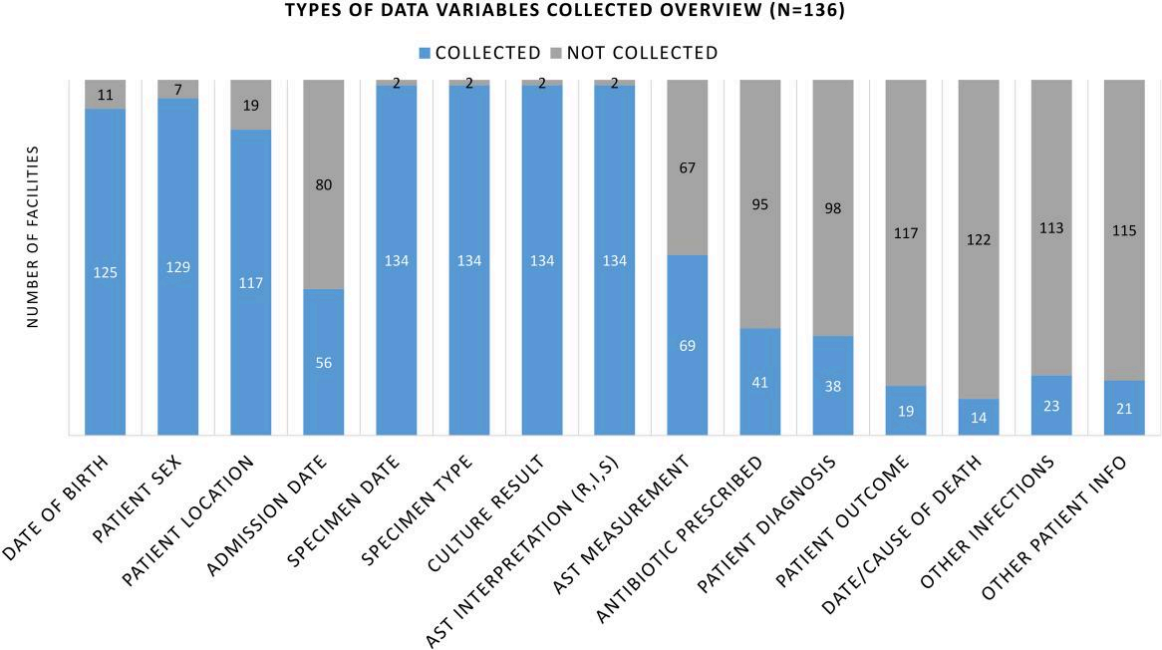
Types of variables collected in 136 laboratories where AST capacity was ascertained. Abbreviations: AST, antimicrobial susceptibility testing; R,I,S, Resistant/Intermediate/Sensitive.

The AMR questionnaire included a variable list that asked the participating laboratories to indicate the types of variables currently being collected (Figure 4). Many laboratories reported collecting variables related to the specimens, such as specimen date (99%, 134/136), type (99%, 134/136), and culture result (99%, 134/136). On the other hand, only a smaller proportion of laboratories reported collecting patient-related or clinical data, such as information on patient outcome (14%, 19/136), diagnoses (30%, 38/136), prescribed antibiotics (30%, 41/136), and admission date (41%, 56/136). The collection of AST interpretation in Resistant/Intermediate/Sensitive (R/I/S) format was observed in most of the laboratories (99%, 134/136), while zone diameter measurements were only seen in half of the laboratories (51%, 69/136).

**Figure 5.**
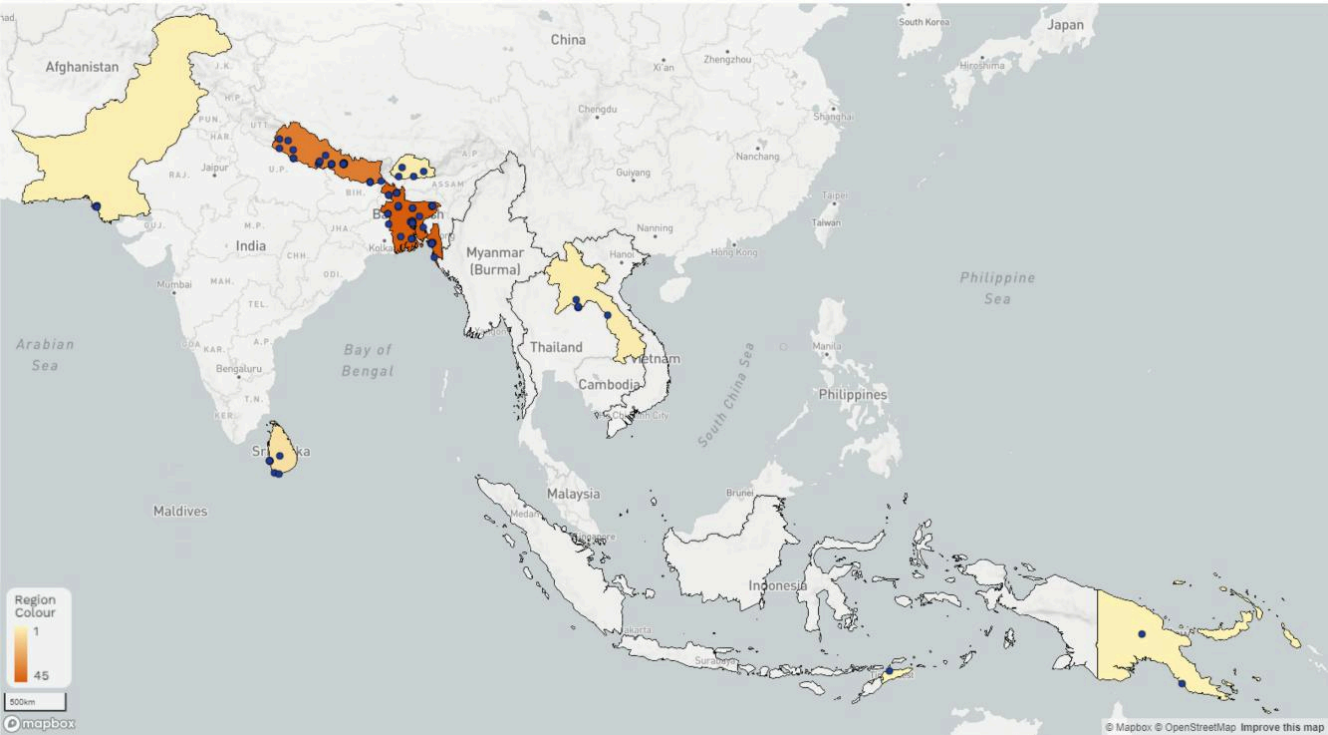
Map of 8 CAPTURA countries where RLQAs were conducted. Location of participating laboratories is indicated with dots. Abbreviations: CAPTURA, Capturing data on Antimicrobial resistance Patterns and Trends in Use in Regions of Asia; RLQA, Rapid Laboratory Quality Assessment.

**Figure 6.**
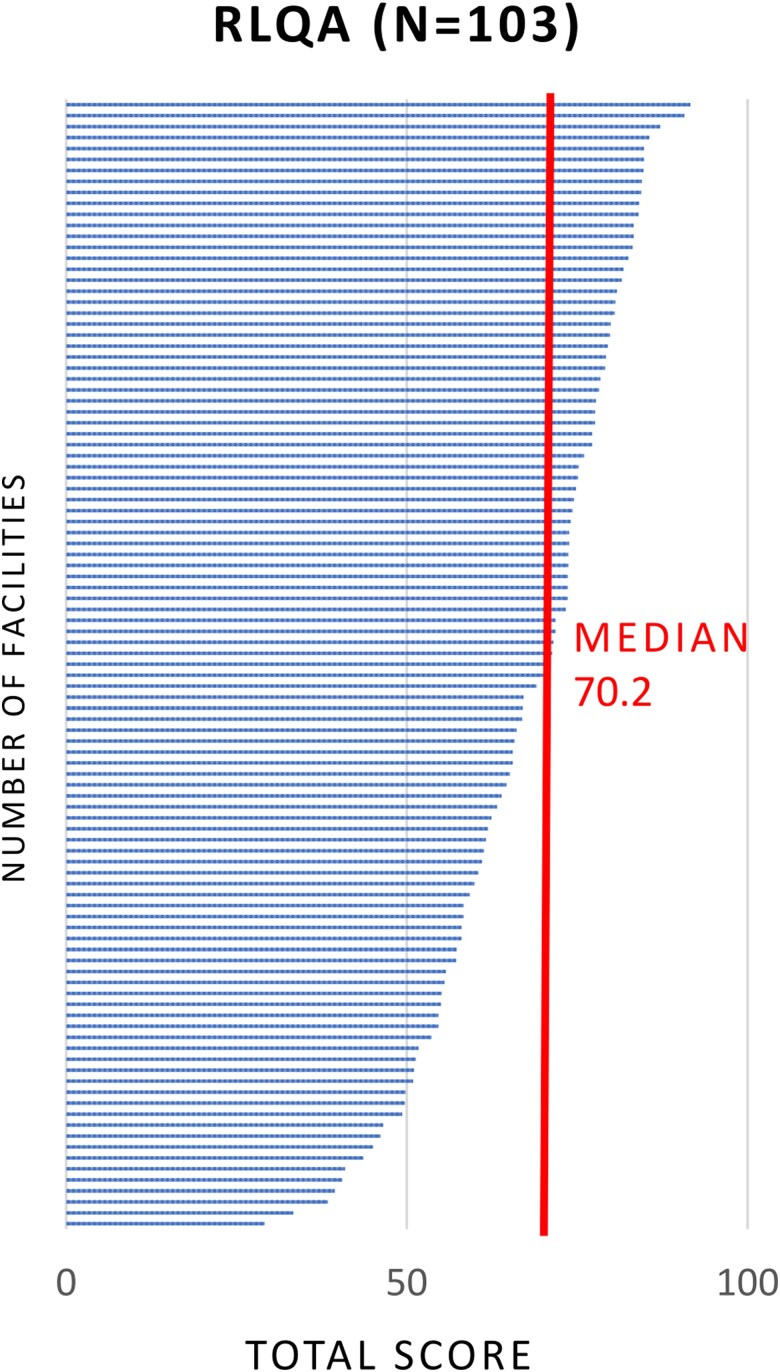
Distribution of total RLQA scores with the median indicated. Abbreviation: RLQA, Rapid Laboratory Quality Assessment.

**Figure 7.**
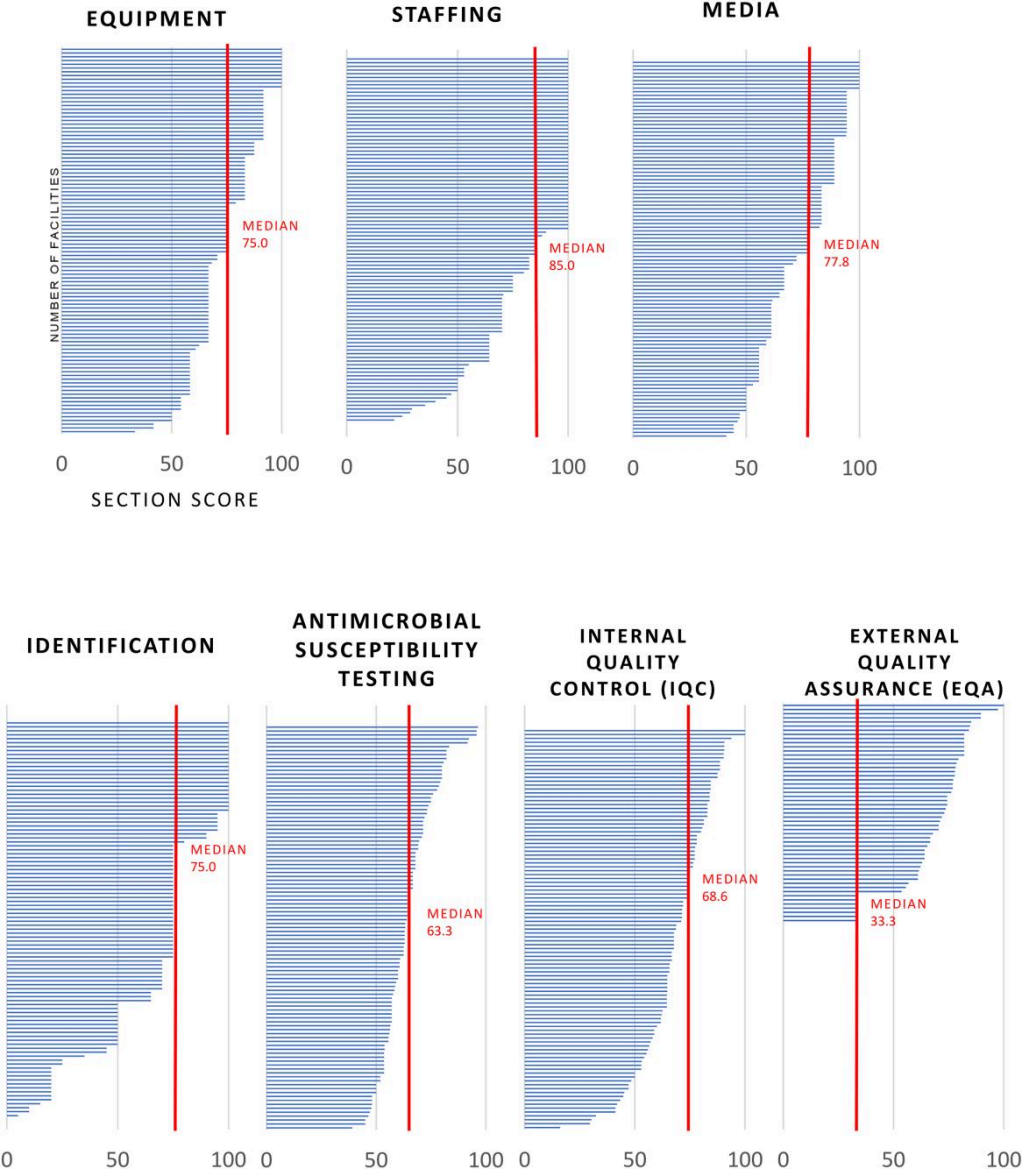
Distribution of RLQA scores per section with median scores indicated. Abbreviation: RLQA, Rapid Laboratory Quality Assessment.

**Figure 8.**
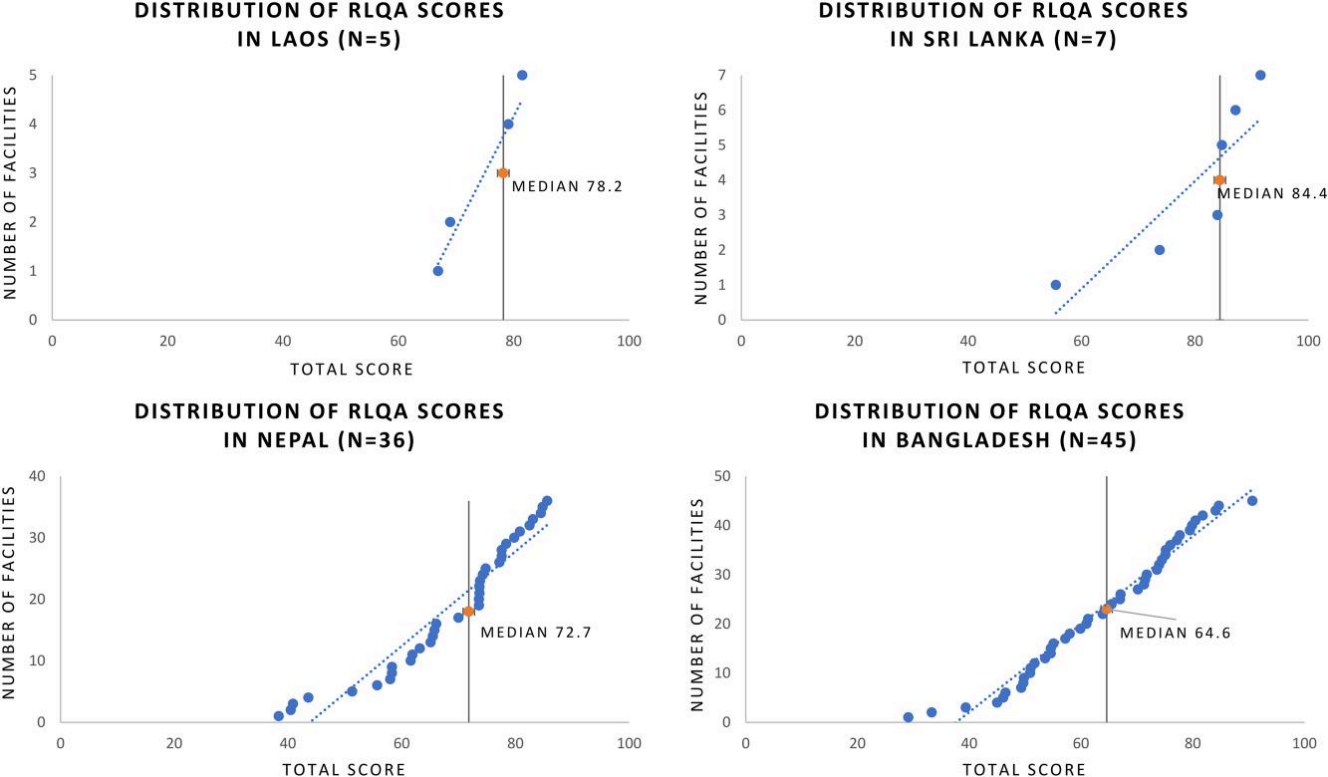
Distribution of total RLQA scores by country. Countries were omitted from figure if <5 assessments were conducted. Abbreviation: RLQA, Rapid Laboratory Quality Assessment.

Once a laboratory's AST capacity was ascertained, the RLQA was conducted in 8 countries (excluding Indonesia). A total of 103 laboratory assessments were collected across Bangladesh (45), Bhutan (4), Laos (5), Nepal (36), Pakistan (3), Papua New Guinea (2), Sri Lanka (7), and Timor-Leste (1) (Figure 5). The highest overall RLQA score from 103 assessments was 91.6 out of 100, while the lowest score was 29.1; the median score was 70.2 (Figure 6). Among the different RLQA sections, the highest median scores from 103 assessments were seen in the Staffing (85.0) and Media (77.8) sections, while the lowest median scores were seen in AST (63.3) and EQA (33.3) (Figure 7). Notably, different levels of capacity were observed within the countries. In Bangladesh, among the 45 RLQA-participating laboratories, the lowest assessment score was 29.1, while the highest score was 90.7 (median: 64.6), resulting in the greatest score gap within a country among all the RLQA-participating countries (Figure 8). While the smallest difference in median scores between countries was noted in the Equipment section (score range between 67.5 and 75.0), the EQA section varied between a score of 0.0 and 84.2.

**Figure 9.**
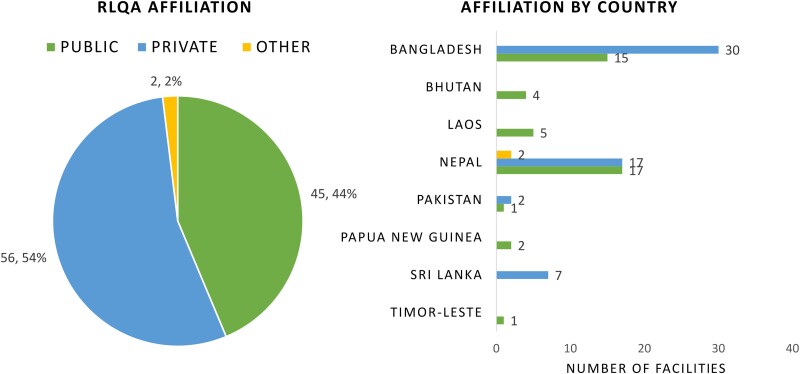
Affiliation of laboratories (public/private/other) participating in RLQA. Abbreviation: RLQA, Rapid Laboratory Quality Assessment.

Fifty-four percent (56/103) of the RLQA-participating laboratories were affiliated with the private sector, while 44% (45/103) were affiliated with the public sector (Figure 9). Private laboratories displayed slightly higher RLQA scores than those of public laboratories. For instance, in Bangladesh, the median overall RLQA score in private laboratories (n = 30) was 67.0, while for public laboratories (n = 15) this was 59.9. Similarly, in Nepal, private laboratories (n = 17) together scored a median of 73.8, while public laboratories (n = 17) scored 65.5. Five countries had a geographic distribution of RLQA-participating laboratories across the country's capital and non-capital regions. Higher RLQA scores were observed in laboratories located within a country's capital than in those located outside of the capital. For example, laboratories located in Dhaka (n = 15) had a median score of 73.6, while laboratories located outside of Dhaka (n = 30) scored 56.2. In Kathmandu, laboratories (n = 18) scored a median of 74.5, while those outside of Kathmandu (n = 18) scored 63.5.

**Figure 10.**
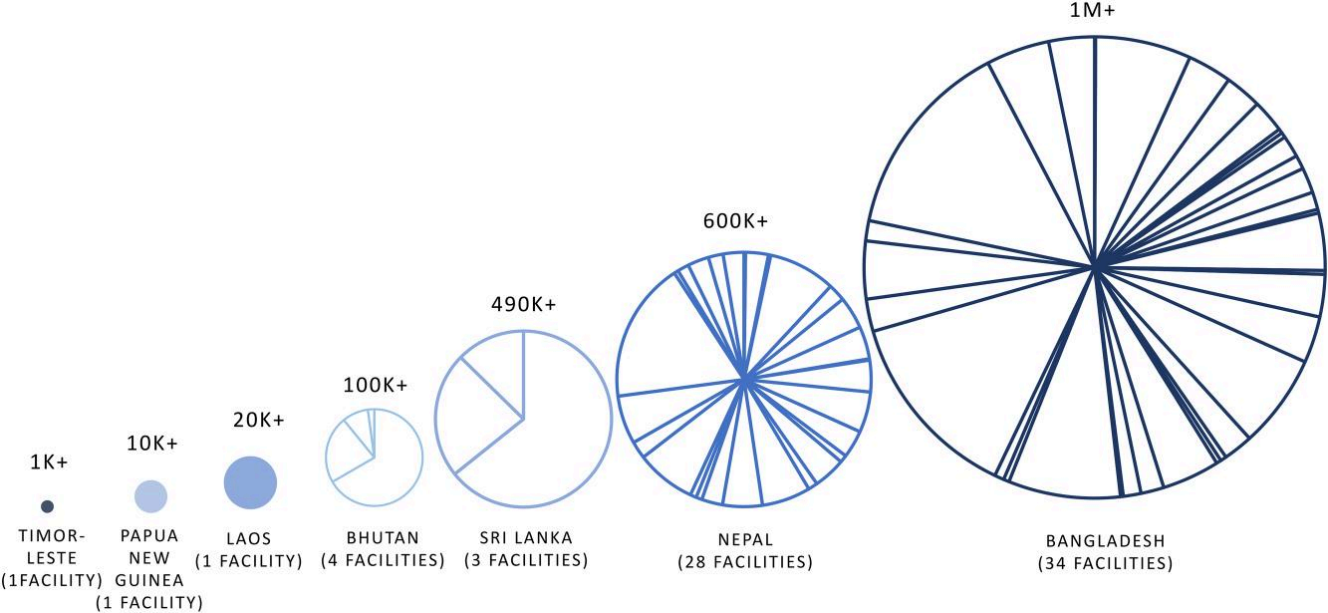
Volume of data shared with project by country. Slices on the pie charts show the number of healthcare facilities where data-sharing agreements were signed (also indicated in brackets), and each slice's size is proportional to the volume of data contributed to the total.

Once the quality of laboratories was assessed through the RLQAs, 7 countries (excluding Pakistan) then proceeded to signing data-sharing agreements with the project. The process leading up to the selection of sites for signing the agreements varied by country, as the implementation strategies and feasibility of activities differed. Ultimately, 72 laboratories across Bangladesh (34), Bhutan (4), Laos (1), Nepal (28), Papua New Guinea (1), Sri Lanka (3), and Timor-Leste (1) shared isolate-level AMR data for retrospective data analyses by the project (Figure 10). The quantity of data shared with the project varied greatly between the countries, ranging from over 1000 records (AST result per isolate) from 1 laboratory in Timor-Leste to over 1 million records from 34 laboratories in Bangladesh.

## DISCUSSION

CAPTURA's project metadata served multiple important purposes during the project. First, they were a tool for effective project implementation, as they influenced the consortium's decisions to prioritize sites and datasets. Second, they served as a guide for understanding facility data once they were collected. In the initial stages of the project, collection of project metadata was emphasized to map out facility data sources (potential data generators and contributors to national and regional data sharing) present in Asia. Adding an additional layer to the mapping of data sources, the questionnaire and assessment results revealed the capacity and quality of laboratories generating AMR data. These results assisted the project in prioritizing which datasets to work on by assessing the level of analyses and interpretation possible; the results also acted as an indicator of the use of the output. Such information also guided the project in providing relevant capacity-building activities and recommendations. As a result, the CAPTURA project was able to prioritize and select the most relevant facilities for further engagement and collaboration. This down-selection process to streamline data collection was labelled in the project as the “funnel approach.”

Beyond the purposes listed above, CAPTURA's project metadata add to the current discourse on the limitations of AMR data in LMICs. There is generally a low volume of AMR data coming from LMICs, as there is both a lack of microbiology laboratories with sufficient AST capacity and a limited amount of testing that is being performed and reported. CAPTURA's metadata collection indicated disk diffusion to be the most used AST method in the region. This is aligned with the fact that disk diffusion is a widespread and conventional method of AST, which may be even more common in resource-limited settings due to its low cost, simpler equipment requirement, and flexibility to test various antibiotics against bacterial species [17, 18]. However, it is important to note that bacterial growth using disk diffusion is time consuming, especially for slow-growing organisms. Further, the growth of bacteria may not occur or be poor/unclear on the media, resulting in human error when measuring growth diameters manually [17]. Therefore, the disadvantages of the disk diffusion method may affect a laboratory's capacity in generating accurate AMR data in a timely manner [17]. Interestingly, however, the small proportion (15%) of laboratories that reported conducting more than 1000 ASTs per month answered that disk diffusion is used to perform AST rather than automated devices, while the laboratories using automated MIC reported conducting fewer than 1000 ASTs per month. This suggests that building laboratory capacity is a multifaceted issue that needs to be addressed first and foremost to generate a larger quantity of data, while also ensuring quality. Previous studies have noted other challenges, such as frequent stockout, irregular supply of consumables and reagents, and overwhelmed human resources as contributing factors behind the limitation of laboratories in the LMICs' AMR surveillance systems [19, 20], all of which were confirmed anecdotally during CAPTURA's interactions with the AMR stakeholders in the countries.

Moreover, many laboratories in Asia are still capturing data on paper, such as logbooks, resulting in scattered or unused data not readily accessible or shareable for analyses. This was evidently seen during the metadata collection period, which led the project to digitize recent historical data and, consequently, to provide hardware installation, training, and troubleshooting sessions. Although more than half of the laboratories (57%) reported keeping both paper and electronic AST data, only up to 3 years' worth of electronic data was available in many laboratories. Only 7 out of 102 laboratories (7%) stored up to 10 years' worth of electronic AST data. This finding suggests that the transition from paper to an electronic data format is an ongoing and recent phenomenon in the region, happening simultaneously as countries explore the introduction of new technologies and equipment through external funding sources such as the FF. Thus, the sustainability of introducing new systems should be carefully vetted and supported [19, 21].

Although a high number of laboratories (74%) reported conducting AMR analyses using their data at the facility level, the project was not able to verify if analyses and interpretation of data were in fact happening. In countries where both public and private laboratories participated in the AMR questionnaire—namely, Bangladesh, Nepal, and Pakistan—there was a greater proportion of private laboratories not sharing data with the Ministry of Health or AMR CC (83%, 35/42) than public laboratories. This may be aligned with previous findings on lack of involvement from private sectors in AMR data sharing [22, 23], which hinders a country's ability to fully understand the emergence of resistance trends across geographies and populations. As hinted by data-variable checklists collected with the AMR questionnaire, there was a lack of clinical and epidemiological data captured along with AMR source data, impeding interpretation and in-depth understanding of the data during analyses. Additionally, relevant metadata, such as quality indicators and denominators, including patient-bed days and population data, were largely absent or challenging to gather. These findings on the lack of clinical, epidemiological, and metadata have already been expressed in previous studies analyzing LMICs' AMR data and is an important issue to address in future efforts to improve AMR surveillance [20, 23].

CAPTURA's experience across several countries in Asia suggests that there is a wide spectrum of capacity and capability of microbiology laboratories within a country and region. Contextually, the CAPTURA project was tasked to work in countries where the surveillance systems are still in the early establishment phase (eg, Papua New Guinea and Timor-Leste), as well as in countries where well-established surveillance networks are ready for expansion (eg, Nepal and Bangladesh). Hence, across the region, varying levels of AMR surveillance capacity were expected from the start of the project, resulting in the project adopting a tailored approach to implement activities aligned with the needs expressed by the country stakeholders [5]. Using CAPTURA's own laboratory assessment tool, the RLQA, the project was able to capture the variability in capacity that currently exists both between countries in the region and between laboratories within the countries. Although CAPTURA's RLQA scores are assessment results developed for project purposes (rather than for formal assessment), they still serve as an indication of current capacity-building needs.

Decision-makers need to consider both gaps/needs and opportunities/initiatives to implement AMR capacity-building activities successfully and sustainably. For instance, the EQA section from the RLQA had the lowest median score compared with other sections (33.3 out of 100) (Figure 7) and displayed the greatest variability between country-specific scores. This finding is aligned with the recent paper on the heterogeneity and lack of EQA programs in Asia [24] and adds to the country-specific information provided in the GLASS-AMR dashboards [25]. Despite understanding the importance of EQA programs and their effectiveness in ensuring laboratory quality, challenges faced by laboratories in LMICs hinder participation in EQA programs. These have been identified as high costs of participation, absence of qualified staff, poor internet access, lack of training opportunities, and difficulty in customs clearance for samples [24]. However, with projects like EQAsia (another FF regional grant), participation in EQAs has become more readily available to counter these challenges [26].

Furthermore, it would be crucial to understand and assess the current capacities and capabilities of healthcare facilities to ascertain their readiness when expanding the AMR surveillance network [27]. CAPTURA's experience in Bangladesh and Nepal suggests that capacity-assessment exercises, such as the RLQA, increase the likelihood of surveillance network expansion as the results can guide decision-makers' discussions on surveillance site selection [21, 28]. For the 2021 data call for GLASS-AMR, 8 healthcare facilities in Bangladesh and 15 facilities in Nepal participated in data submission [25]. With CAPTURA's RLQA results highlighting a list of laboratories with sufficient capacity and availability of relevant data, country stakeholders have been encouraged to follow up and reassess (verifying the RLQA findings) prior to inclusion in the surveillance network. Moreover, as RLQA results suggest capacity differences between private and public laboratories, as well as between laboratories located in a country's capital and non-capital regions, it is important to widen the selection of sentinel sites and data sources included to ensure better representation in national surveillance systems. Despite the challenges in standardizing collection processes and data-sharing platforms, gathering more complete and representative data would be essential to improve our current understanding of AMR and its true picture in LMICs [23].

There were several limitations to CAPTURA's project metadata. First, CAPTURA's questionnaires and laboratory assessment were project-specific tools developed for project-specific purposes, and therefore these tools were not validated before use. The tools were not deployed to generate significant study findings on capacity and availability of data; rather, the aim was to take a snapshot at the time of project initiation to have findings guide the CAPTURA project implementation. Therefore, the metadata collection was an inherently pragmatic approach to sample and gather information fitting to a specific context. As a result, questionnaire and laboratory assessments were conducted once per facility, and responses were not verified. Additionally, information captured and presented in this paper is now likely to be outdated, as it does not account for the numerous capacity-building and infrastructure-strengthening activities in the last few years since the point of collection. Second, due to the unforeseen COVID-19 pandemic, travel became increasingly challenging for the CAPTURA consortium members and country team [11]. Therefore, on top of the already pragmatic approach, virtual and convenient means to data collection were adopted to ensure the safety of the country team and staff of the participating sites [11]. For some countries, the list of healthcare facilities for metadata collection was altered to account for travel restrictions and lockdown orders. Several countries' questionnaires and laboratory assessments varied greatly in dates of administration, as travel was often delayed. Due to these limitations, the findings of CAPTURA's project metadata may not be generalizable nor best representing the true and most up-to-date picture in the region. Nevertheless, this paper illustrates the usefulness of gathering project metadata to guide project implementation, enabling a funnel approach to identify data sources, assessing data quality, and selecting sites for data collection. In addition to project purposes, using the gathered information with these limitations in mind, it can serve as an indication of current capacity-building needs and be used in guiding activities by relevant stakeholders to enhance surveillance systems.

## Supplementary Data

Supplementary materials are available at *Clinical Infectious Diseases* online. Consisting of data provided by the authors to benefit the reader, the posted materials are not copyedited and are the sole responsibility of the authors, so questions or comments should be addressed to the corresponding author.

## Supplementary Material

ciad634_Supplementary_Data

## Notes

***Disclaimer.*** The views expressed here are those of the authors and not necessarily those of the UK Department of Health and Social Care or its management agent, Mott MacDonald; and, International Vaccine Institute.

***Author Contributions.*** M. H., W. M., D. M. A., J. S., and F. M. conceived the original concept and design of the CAPTURA project. H. S. J., A. Shaw., N. P., P. G., J-H. K., A. Shaikh., H. S., W. M., and M. H. provided instrumental efforts as country point of contacts implementing country activities with in-country teams. H. S. J., C. Y., K. K. C., K. P., A. Cho., M. J. S., E. E., K. D. P., S. S., A. T. A., J. S., N. P., and M. H. analyzed and interpreted data, producing summary reports with recommendations for country and facility stakeholders. H. S. J., M. J. S., G. P., GH. J., E. L. P., H-W. S., K. A., B. E. W. T., A. Clark., J. S., and D. M. A. supported setting up data warehouse and visualization tools. B. D., H. Y., J. H., S. K., N. P., and M. H. ensured administrative functions by overseeing project finance and human resources. M. H. made substantial contributions to the concept of this paper and revised for important intellectual content. H. S. J. wrote the manuscript.

***Acknowledgments.*** The collection of CAPTURA's project metadata would not have been possible without the country coordinators and teams. The authors express their gratitude to the following individuals (in alphabetical order) for their support: Abul Hasnat, Amit Bhandari, Bouahome Phommalad, Delfim Costa, Hapudeniya Muditha, Hasini Banneheke, Hridika Talukdar Barua, Mohammad Julhas Sujan, Kulunu Ranasinghe, M. Naeem Akhtar, Manish Gautam, Michelle Hau’ofa, Nashmia Mahmood, Nurul Huda, Pema Yangzom, Piyumaka Udara Peiris, Quyen Nguyen, Reena Mukhiya, Smriti Kattel, and Truong Le Van Ngoc.

***Supplement sponsorship.*** This article appears as part of the supplement “Capturing Data on Antimicrobial Resistance Patterns and Trends in Use in Regions of Asia (CAPTURA),” funded by the Department of Health and Social Care's Fleming Fund using UK aid.

***Financial support.*** This work was supported by the Fleming Fund regional grants (grant numbers FF10-135 and FF11-139). The Fleming Fund is a UK aid investment to support surveillance of antimicrobial resistance in low- and middle-income countries in Africa and Asia. The regional grants program is managed by Mott MacDonald Ltd, the Fleming Fund Grants Management Agent.
