# Supplementary material for: Methodological Approach to Identify and Expand the Volume of Antimicrobial Resistance (AMR) Data in the Human Health Sector in Low- and Middle-Income Countries in Asia: Implications for Local and Regional AMR Surveillance Systems Strengthening
Source: Clin Infect Dis. 2023 Dec 20;77(Suppl 7):S507–18. doi: 10.1093/cid/ciad634 (PMC10732564; doi:10.1093/cid/ciad634)
Supplement: ciad634_Supplementary_Data [file ciad634_supplementary_data.zip › 9. CAPTURA Selected variables for visualisation_revised.pdf]

CAPTURA: variables selected for visualisation

AMR QUESTIONNAIRE
Types of bacterial cultures performed (specimens obtained from blood/cerebrospinal fluid/genitals/respiratory tract/soft tissue and body fluids/ stool/ urine)
Antimicrobial susceptibility testing (AST)conducted & average number per month
Methods of AST (automated system/manual/E-test/disk diffusion)
Records of AST results (paper/electronic)
If electronic, software used to record AST results (LIS/ WHONET/ MIC instrument software)
Number of years of AST records available for
Analysis and sharing of AST results with other institution(s)
Variables included in facility database/records
**AMU QUESTIONNAIRE**
Clinical departments supplied
Certification
Guidelines for storing, stocking and dispensing
Number and qualification of staff
Periodic training
Sourcing of antimicrobials
Records of antimicrobials distributed/sold (electronic/paper based)
Software used
Years recorded antimicrobials distributed/sold
Format of data exported
Data (raw or analysed) on antimicrobials dispensed/ sold ever sent to/shared with another organisation or facility (name, content and frequency)
Prescription/over the counter sales
Which drugs are obtained WITHOUT prescription
Patient diagnosis on prescriptions (or in similar patient linked document)
Pharmacy access to the laboratory culture results for the patient
Pre-analysed data/information on what antimicrobials are dispensed/sold
Variables included in facility database/records
**RAPID LABORATORY QUALITY ASSESSMENT**
Overall scores of the RLQA
Per- section score of RLQA
Equipment – presence/ status of use of 12 key lab equipment
Staff – number of senior staff & qualification, number of bench staff & status of training
Media – media used in lab, source of media plates (purchased and in-house)
Identification – methods of performing blood cultures, tests conducted in lab, specificity of reporting
Antimicrobial Susceptibility Testing – methods of performance, guidelines followed, use of Mueller-Hinton plates, updates of breakpoints
Internal Quality Control – use of standard strains (ATCC/NCTC), calibration of incubator, steps taken for media quality, use and status of SOPs
External Quality Assurance – participation in EQA, names/providers of EQA, pathogens included in test panel
Visual inspection – visual checks of media, pathogen identification algorithm, temperature charts, breakpoints used (CLSI), data capture/ storage
