# Supplementary material for: Methodological Approach to Identify and Expand the Volume of Antimicrobial Resistance (AMR) Data in the Human Health Sector in Low- and Middle-Income Countries in Asia: Implications for Local and Regional AMR Surveillance Systems Strengthening
Source: Clin Infect Dis. 2023 Dec 20;77(Suppl 7):S507–18. doi: 10.1093/cid/ciad634 (PMC10732564; doi:10.1093/cid/ciad634)
Supplement: ciad634_Supplementary_Data [file ciad634_supplementary_data.zip › Appendix 1. CAPTURA AMR Laboratory Questionaire.pdf]

CAPTURA

Capturing data on Antimicrobial resistance Patterns and Trends in Use in Regions of Asia

QUESTIONS FOR LABORATORIES

The information provided will assist the CAPTURA consortium and Ministry of Health in each country to understand the AMR data available at each facility, the methods used to collect it, format of the stored data, and additional indicators that will assist the consortium in prioritizing facilities with the most relevant datasets for AMR surveillance. The provided information will also be used to map where AMR data exists in the country to further the Ministry of Health's knowledge for strategic planning.

This questionnaire should take about 5 – 10 minutes. Thank you for your time in advance.

**By participating in this laboratory questionnaire developed by the CAPTURA consortium (as a part of the Fleming Fund Grants Programme managed by Mott MacDonald), you are agreeing for your responses to be stored and utilized for CAPTURA project activities. The responses may be shared with other Fleming Fund stakeholders and partners for purposes relating to Fleming Fund activities. For details on Mott MacDonald's privacy policy, please see the link to the website: www.mottmac.com/privacy-policy.*

** I agree and understand the above statements* ☐

*** Date of completion (dd/mm/yyyy)**	
*** Name of person completing this form**	
*** Email of person completing this form**	
**Phone number of person completing this form**	
*** Name of laboratory (or hospital in which the laboratory is based)**	
*** Country**	
*** City/ town**	
**State**	
**Province**	
**District**	

* indicates required fields

1	* What type of facility is this lab?	Public laboratory ☐	Private laboratory ☐	Military laboratory ☐	Other ☐ ________			
2	Is your laboratory located within a hospital?	Yes ☐	No ☐					
3	* What types of culturing does your laboratory do? Select all that apply.	Blood ☐	Cerebro-spinal fluid (CSF) ☐	Genital ☐	Respira-tory Samples ☐	Soft tissue and body fluids ☐	Stool ☐	Urine ☐
		Other ☐ _________________						
4	* Do you conduct Antimicrobial Susceptibility Testing (AST)?	Yes ☐ (Please continue)	No ☐ (Proceed to Question 17)	Don't know ☐ (Proceed to Question 17)				
5	* How is Antimicrobial Susceptibility Testing (AST) performed? Select all that apply.	Automated MIC (Vitek, Microscan, Phoenix, other) ☐	Manual MIC determination ☐	Etest ☐	Disk diffusion ☐	Other ☐ ____________		
6	Approximately how many Antimicrobial Susceptibility Testing (AST) do you perform during an average month?	1-10 ☐	11-50 ☐	51-100 ☐	101-1,000 ☐	More than 1,000 ☐	Don't know ☐	
7	* Do you record the Antimicrobial Susceptibility Testing (AST) result?	Yes ☐ (Please continue)	No ☐ (Proceed to Question 17)					
8	* If yes, how are the Antimicrobial Susceptibility Testing (AST) results recorded?	Paper (logbook) ☐ (please answer Question 9 and then proceed to Question 12)	Electronic ☐ (please answer Question 9 and continue to Question 10)	Both ☐ (please answer Question 9 and continue to Question 10)				
9	* For how many years has your facility recorded Antimicrobial Susceptibility Testing (AST) results (both paper and electronic)?	______________ years	Don't know ☐					

10	* If electronic, what software is used to record the Antimicrobial Susceptibility Testing (AST) results? Select all that apply.	Laboratory Information System ☐ Name: ______________	WHONET ☐	MIC instrument software ☐ Name: ____________	Other ☐ ____________				
11	* How many years of data (electronic format only) does the antimicrobial resistance (AMR) dataset contain?	< 1 year ☐	1-3 years ☐	3-10 years ☐	More than 10 years ☐	Don't know ☐			
12	* Do you analyse your antimicrobial resistance (AMR) data?	Yes ☐	No ☐ (Proceed to Question 15)	Don't know ☐ (Proceed to Question 15)					
13	What systems do you use to analyse AMR data? Select all that apply.	Manual data analysis ☐	WHONET ☐	Excel ☐	Laboratory Instrument ☐	Laboratory Information System ☐	Other ☐ _______		
14	How often do you produce reports? Select all that apply.	Daily ☐	Weekly ☐	Monthly ☐	Quarterly ☐	Every 6 months ☐	Yearly ☐	Irregularly ☐	Don't know ☐
15	* Are isolate-level antimicrobial resistance (AMR) data ever sent to another organisation or facility?	Yes ☐ (Please continue)	No ☐ (Proceed to Question 17)	Don't know ☐ (Proceed to Question 17)					
16	*If yes, where is the data sent?								
17	Does your facility have reliable internet connectivity?	Yes ☐ (Please continue)	No ☐ (Proceed to Question 19)						
18	If yes, what is the bandwidth? (Mbps)	_______Mbps	Don't know ☐						
19	* Does your facility have an IT contact person?	Yes ☐ (Please continue)	No ☐ (Proceed to next page)	Don't know ☐ (Proceed to next page)					
20	If yes, are you able to share contact details?	Able to share ☐<br>Name:<br>Email/Skype/Phone number:	Unable to share ☐						

Please select all data variables that are included in the database/ records at your facility.	
SAMPLE AMR DATA VARIABLES	Variable Collected
*** Routine (Priority CAPTURA Variables)**	
Sample Origin (Human/Animal/Food)	
Date of Birth/ Age	
Sex	
Patient location (ward/clinic)	
Healthcare Facility Admission Date (if inpatient)	
Healthcare Facility Date of Visit (if outpatient)	
Specimen Date	
Specimen Type	
Culture Result (organism isolated)	
AST Interpretation (R, I, S)	
AST Measurement (disk diffusion zone diameter/MIC value)	
**Specialised/Targeted (Optional CAPTURA Variables)**	
Antibiotics Prescribed After Specimen Collection	
Diagnosis (after laboratory results provided)	
Patient Outcome	
Date and Cause of Death (if applicable)	
Additional/Recurrent Isolates/Infections	
Additional Patient Information (e.g. change in initial therapy, date of discharge, comorbidities, date of discharge, etc.)	
