# Supplementary material for: Methodological Approach to Identify and Expand the Volume of Antimicrobial Resistance (AMR) Data in the Human Health Sector in Low- and Middle-Income Countries in Asia: Implications for Local and Regional AMR Surveillance Systems Strengthening
Source: Clin Infect Dis. 2023 Dec 20;77(Suppl 7):S507–18. doi: 10.1093/cid/ciad634 (PMC10732564; doi:10.1093/cid/ciad634)
Supplement: ciad634_Supplementary_Data [file ciad634_supplementary_data.zip › Appendix 2. CAPTURA AMU Pharmacy Questionnaire.pdf]

CAPTURA

Capturing data on Antimicrobial resistance Patterns and Trends in Use in Regions of Asia

QUESTIONS FOR PHARMACIES/DISPENSARIES

The information provided will assist the CAPTURA consortium and Ministry of Health understand the antimicrobial use (AMU) data available at each facility, the methods used to collect it, format of the stored data, and additional indicators that will assist the consortium in prioritizing facilities with the most relevant datasets for AMR surveillance. The provided information will also be used to map where AMU data exists in the country to further the Ministry of Health's knowledge for strategic planning.

This questionnaire should take about 10-15 minutes. Thank you for your time in advance.

**By participating in this Antimicrobial Use questionnaire developed by the CAPTURA consortium (as a part of the Fleming Fund Grants Programme managed by Mott MacDonald), you are agreeing for your responses to be stored and utilized for CAPTURA project activities. The responses may be shared with other Fleming Fund stakeholders and partners for purposes relating to Fleming Fund activities. For details on Mott MacDonald's privacy policy, please see the link to the website: www.mottmac.com/privacy-policy.*

** I agree and understand the above statements* ☐

*** Date of completion (dd/mm/yyyy)**	
*** Name of person completing this form**	
*** Email of person completing this form**	
**Phone number of person completing this form**	
*** Name of pharmacy/dispensary (or hospital where it is based)**	
*** Country**	
*** City/ town**	
**State**	
**Province**	
**District**	

****Please make sure you have marked your consent above****

1.	* What type of facility is this pharmacy/dispensary?	Public Pharmacy/ Dispensary ☐	Private Pharmacy/ Dispensary ☐	Other ☐ ________________	
2.	*Is your pharmacy/ dispensary located within a hospital/health centre?	Yes ☐	No ☐ (Proceed to Question 5)		
3.	If yes, what is the name of the hospital/ health centre?	____			
4.	If yes, which departments does your pharmacy/dispensary provide pharmaceuticals to? Select all that apply.	In-Patient Department(s) ☐	Out-Patient Department(s) ☐	Emergency Department(s) ☐	Other ☐ ____________
5.	*What type of certification does the pharmacy hold? Please provide details on the certification (e.g., name of certification, when received, who provided)	____			
6.	*Approximately how many staff work in your pharmacy/ dispensary?	______________________			
7.	*Of those staff working in your pharmacy/ dispensary, approximately how many are certified pharmacists?	______________________	Don't know ☐		
8.	*Do staff in your pharmacy follow a procedural guideline for dispensing drugs including antimicrobials?	Yes ☐	No ☐ (Proceed to Question 10)	Don't know ☐ (Proceed to Question 10)	
9.	If yes, please provide details (e.g., name of guideline, version, etc)	____			
10.	*Do staff in your pharmacy follow a procedural guideline for storing and stocking drugs including antimicrobials?	Yes ☐	No ☐ (Proceed to Question 12)	Don't know ☐ (Proceed to Question 12)	
11.	If yes, please provide details (e.g., name of guideline, version, etc)	____			
12.	*Do staff receive periodic training to review and follow procedural guidelines mentioned above?	Yes ☐	No ☐	Don't know ☐	
13.	*Where do you source antimicrobials from? Select all that apply.	Ministry of Health / Government Drug authority ☐	Private company/ distributors ☐	Other ☐ ________________	

**14.**	***Are the antimicrobials distributed/sold at this facility recorded?**	Yes ☐		No ☐<br>(Proceed to Question 22)		
**15.**	***In what format are these recorded?**	Paper<br>☐<br>(please answer Question 16 and proceed to Question 22)	Electronic<br>☐<br>(Proceed to Question 17)	Both<br>☐<br>(please answer Question 16 and proceed to 17)		
**16.**	**How many years has your facility recorded antimicrobials distributed on paper?**<br>Please ignore this question if you have chosen "Electronic"	________________years		Don't know ☐		
**17.**	***If electronic, what software is used to record the antimicrobials distributed?** Select all that apply.	mSupply ☐	AdenBox☐	Excel ☐	Other ☐<br>__________	
**18.**	**How many years has your facility recorded antimicrobials distributed/ sold using the selected software?** Answer all that apply.	mSupply<br>______years	AdenBox<br>______years	Excel<br>______years	Other, as specified above<br>______years	Don't know<br>☐
**19.**	***Who procured the software used to record antimicrobials distributed/ sold?** Select all that apply.	Government/ Ministry of Health<br>☐	Facility/ Hospital<br>☐	Other ☐<br>_________________		
**20.**	***Using the selected software, have you previously exported your data on antimicrobials distributed/ sold?**	Yes ☐	No ☐<br>(Proceed to Question 22)	Don't know ☐<br>(Proceed to Question 22)		
**21.**	**If yes, in what format can data be exported?** Select all that apply.	Txt (Text File)<br>☐	xls/ xlsx (Excel)<br>☐	csv (Comma-separated value) ☐	Other ☐<br>____________	
**22.**	***Do patients need a prescription (Rx) to obtain antibiotics from your facility?**	Yes ☐<br>(Proceed to Question 24)	No ☐<br>(Proceed to Question 26)	Sometimes ☐		
**23.**	**If chosen "Sometimes", what drugs are obtained WITHOUT prescription?**	____				
**24.**	***If chosen "Yes" or "Sometimes", does the pharmacy retain a copy of the prescription?**	Yes ☐	No ☐	Sometimes ☐	Don't know<br>☐	

**25.**	*Are there records of patient diagnosis on prescriptions (or in similar patient linked document)?	Yes ☐	No ☐	Sometimes ☐	Don't know ☐
**26.**	*Does the pharmacy have access to the laboratory culture results for the patient?	Yes ☐	No ☐ (Proceed to Question 28)	Sometimes ☐	Don't know ☐ (Proceed to Question 28)
**27.**	If chosen "Yes" or "Sometimes", please provide more details (e.g., how often do you have access, how do you access the results)	______________________________________			
**28.**	*Do you analyse data/information on what antimicrobials are dispensed/sold?	Yes ☐	No ☐ (Proceed to Question 31)	Don't know ☐ (Proceed to Question 31)	
**29.**	If yes, how is the analysis on antimicrobials dispensed/sold used? (e.g., to check stock)	______________________________________			
**30.**	If yes, what software do you use to analyse the data/information? Select all that apply.	Manual data analysis ☐	Excel ☐	Inventory software or other ☐ __________	

**31.**	*How often do you produce reports using data (raw or analysed)? Select all that apply.	Daily ☐	Weekly ☐	Monthly ☐	Every 6 months ☐	Yearly ☐	Don't know ☐	Other ☐ _____

**32.**	*Is the data (raw or analysed) on antimicrobials dispensed/ sold ever sent to another organisation or facility?	Yes ☐	No ☐ (Proceed to Question 35)	Don't know ☐ (Proceed to Question 35)		
**33.**	If yes, where is the data sent?	______________________________________				
**34.**	If yes, how often have you sent data?	Daily ☐	Weekly ☐	Monthly ☐	Yearly ☐	Other ☐ _______
**35.**	*Does your facility have reliable internet connectivity?	Yes ☐	No ☐			
**36.**	*Does your facility have an IT contact person?	Yes ☐	No ☐ (Proceed to next page)	Don't know ☐ (Proceed to next page)		
**37.**	If yes, are you able to share contact details?	Able to share ☐<br>Name:<br>Email/Skype/Phone number:	Unable to share ☐			

Please select (✓) all data variables that are included in the database/ records at your facility.	
SAMPLE AMU DATA VARIABLES	Variable Collected (✓)
* Routine (Priority CAPTURA Variables)	
Patient Age	
Patient Sex	
Date of Prescription	
Department (OPD, IPD, Emergency)	
Type of Drug (Drug Class)	
Ingredients	
Strength of Drug	
Formulation Type	
Route of Administration	
Product Name	
Manufacturer	
Pack Size Unit/ Number of Doses Distributed	
Daily Defined Doses (DDD)	
Anatomical Therapeutic Chemical Classification System (ATC codes)	
* Specialised/ Targeted (Optional CAPTURA Variables)	
Indication for Prescription/ Diagnosis	
MDR Risk	
Product Origin	
Brand Name or Generic	
Previous Antimicrobial Prescriptions	
Change to Initial Therapy	

****Please make sure you have marked your consent on the first page****
