# Supplementary material for: Methodological Approach to Identify and Expand the Volume of Antimicrobial Resistance (AMR) Data in the Human Health Sector in Low- and Middle-Income Countries in Asia: Implications for Local and Regional AMR Surveillance Systems Strengthening
Source: Clin Infect Dis. 2023 Dec 20;77(Suppl 7):S507–18. doi: 10.1093/cid/ciad634 (PMC10732564; doi:10.1093/cid/ciad634)
Supplement: ciad634_Supplementary_Data [file ciad634_supplementary_data.zip › Appendix 3. CAPTURA Questionnaire deployment method.pdf]

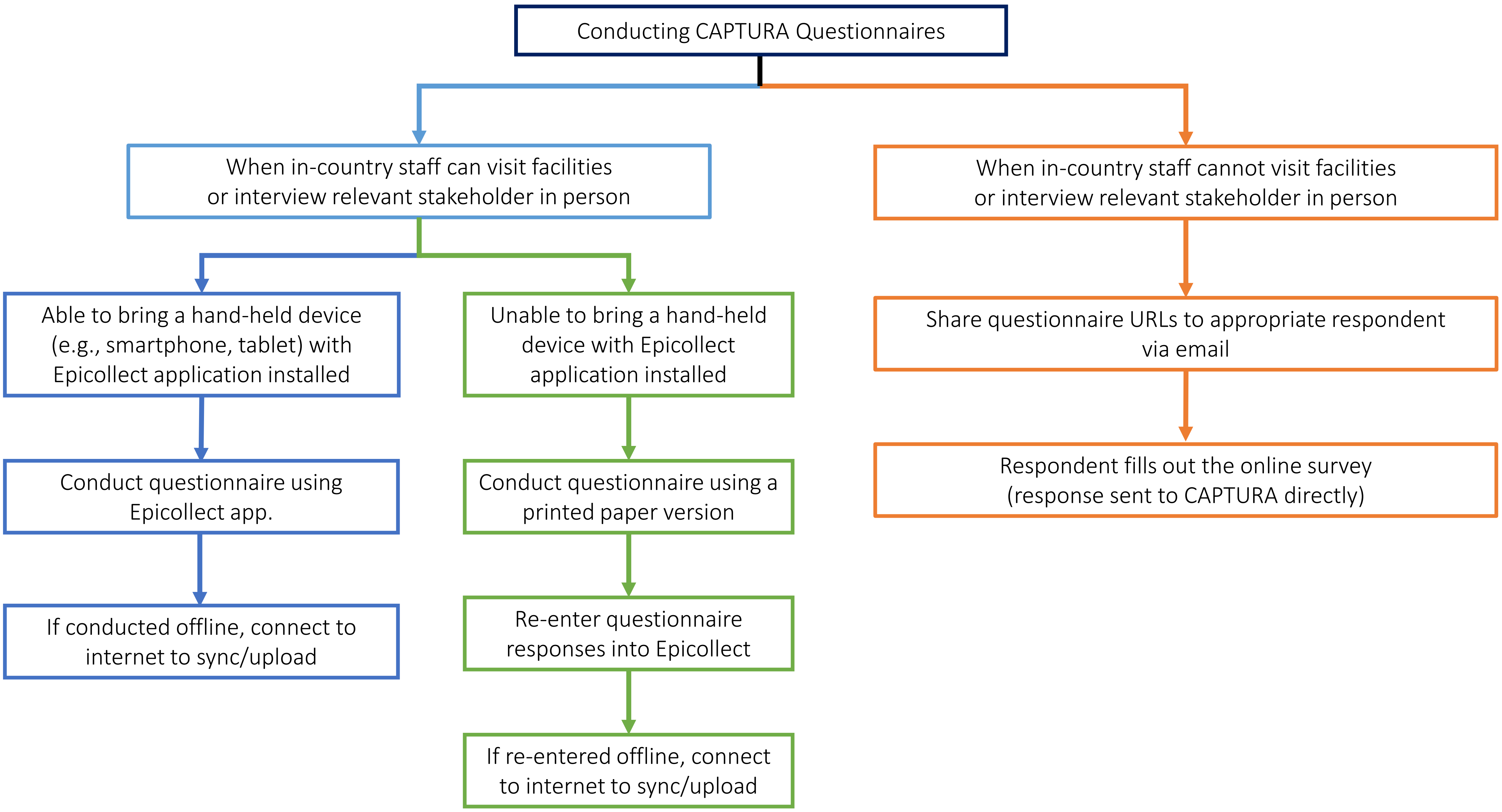
Conducting CAPTURA Questionnaires
When in-country staff can visit facilities or interview relevant stakeholder in person
When in-country staff cannot visit facilities or interview relevant stakeholder in person
Able to bring a hand-held device (e.g., smartphone, tablet) with Epicollect application installed
Unable to bring a hand-held device with Epicollect application installed
Share questionnaire URLs to appropriate respondent via email
Conduct questionnaire using Epicollect app.
Conduct questionnaire using a printed paper version
Respondent fills out the online survey (response sent to CAPTURA directly)
If conducted offline, connect to internet to sync/upload
Re-enter questionnaire responses into Epicollect
If re-entered offline, connect to internet to sync/upload
