# Supplementary material for: Methodological Approach to Identify and Expand the Volume of Antimicrobial Resistance (AMR) Data in the Human Health Sector in Low- and Middle-Income Countries in Asia: Implications for Local and Regional AMR Surveillance Systems Strengthening
Source: Clin Infect Dis. 2023 Dec 20;77(Suppl 7):S507–18. doi: 10.1093/cid/ciad634 (PMC10732564; doi:10.1093/cid/ciad634)
Supplement: ciad634_Supplementary_Data [file ciad634_supplementary_data.zip › Appendix 4. CAPTURA RLQA and scoring guide.pdf]

Methodology for Grading of the Laboratory Assessment

The tool will be placed into an electronic format, where grading will be automated. This document provides the methodology behind the grading.

I. Grading by Section and the overall lab quality grade

Each section will hold a weight for the overall grade. The final score will be out of 100%, with each of the follow section weights:

Equipment (10%)	Identification (15%)
Staffing (20%)	Media (10%)
AST (20%)	IQC (15%)
EQAS (10%)	

The associated data collected will be linked to the lab name so that the we can easily utilize the information for map layers and also the overall score will be used to determine how much weight should be put on the data set during analysis.

II. Grading within each section

Each question will have a number of points associated with it. There are certain questions that will hold more weight in a given section if they are highly critical to quality. We have placed the number of points in the right most column. Some questions are just for collecting information for map generation and will not be graded.

CAPTURA

Capturing data on Antimicrobial resistance Patterns and Trends in Use in Regions of Asia

Rapid Laboratory Quality Assessment Tool for AMR

ID of dataset being graded	
Name of person conducting grading	
Date of grading	
Name of laboratory being graded	
Country	
City, State/Department/Province	

Score Card

Section	A.<br>% of total	B.<br>Points Earned	C.<br>Points in Section	D.<br>B / C	E<br>A*D*100
Equipment	10%		24		%
Staffing	20%		20		%
Media	10%		18		%
Identification	15%		20		%
AST	20%		30		%
IQC	15%		38		%
EQAS	10%		78		%
**TOTAL**	**100%**				**%**

Equipment (___/24)

1	Bunsen burner/Spirit lamp/electric burner	Present?	Yes ☐		No ☐	No score
2		Functional/Operational?	Yes ☐		No ☐	1 Point / 0 Points
3		In use for last 3 years?	Yes ☐	No ☐	Don't know ☐	1 Point / 0 Points / Remove
4	Refrigerator	Present?	Yes ☐		No ☐	No score
5		Functional/Operational?	Yes ☐		No ☐	1 Point / 0 Points
6		In use for last 3 years?	Yes ☐	No ☐	Don't know ☐	1 Point / 0 Points / Remove
7	Incubator	Present?	Yes ☐		No ☐	No score
8		Functional/Operational?	Yes ☐		No ☐	1 Point / 0 Points
9		In use for last 3 years?	Yes ☐	No ☐	Don't know ☐	1 Point / 0 Points / Remove
10	Weighing balance	Present?	Yes ☐		No ☐	No score
11		Functional/Operational?	Yes ☐		No ☐	1 Point / 0 Points
12		In use for last 3 years?	Yes ☐	No ☐	Don't know ☐	1 Point / 0 Points / Remove
13	Microscope	Present?	Yes ☐		No ☐	No score
14		Functional/Operational?	Yes ☐		No ☐	1 Point / 0 Points
15		In use for last 3 years?	Yes ☐	No ☐	Don't know ☐	1 Point / 0 Points / Remove
16	-20°C freezer	Present?	Yes ☐		No ☐	No score
17		Functional/Operational?	Yes ☐		No ☐	1 Point / 0 Points
18		In use for last 3 years?	Yes ☐	No ☐	Don't know ☐	1 Point / 0 Points / Remove
19	-80°C freezer	Present?	Yes ☐		No ☐	No score
20		Functional/Operational?	Yes ☐		No ☐	1 Point / 0 Points
21		In use for last 3 years?	Yes ☐	No ☐	Don't know ☐	1 Point / 0 Points / Remove
22	Water bath	Present?	Yes ☐		No ☐	No score
23		Functional/Operational?	Yes ☐		No ☐	1 Point / 0 Points

24		In use for last 3 years	Yes ☐	No ☐	Don't know ☐	1 Point / 0 Points / Remove
25	pH Meter	Present?	Yes ☐	No ☐		No score
26		Functional/Operational?	Yes ☐	No ☐		1 Point / 0 Points
27		In use for last 3 years?	Yes ☐	No ☐	Don't know ☐	1 Point / 0 Points / Remove
28	Laminar flow	Present?	Yes ☐	No ☐		No score
29		Functional/Operational?	Yes ☐	No ☐		1 Point / 0 Points
30		In use for last 3 years?	Yes ☐	No ☐	Don't know ☐	1 Point / 0 Points / Remove
31	Biosafety cabinet	Present?	Yes ☐	No ☐		No score
32		Functional/Operational?	Yes ☐	No ☐		1 Point / 0 Points
33		In use for last 3 years?	Yes ☐	No ☐	Don't know ☐	1 Point / 0 Points / Remove
34	Autoclave	Present?	Yes ☐	No ☐		No score
35		Functional/Operational?	Yes ☐	No ☐		1 Point / 0 Points
36		In use for last 3 years?	Yes ☐	No ☐	Don't know ☐	1 Point / 0 Points / Remove

Staff (__ / 20)

37	Senior staff	How many senior staff have clinical microbiology qualification?	1+☐	0 ☐	Don't know ☐	4 points / 0 points / Remove
38		What qualification(s) do(es) the clinical microbiologist(s) have?	Qualification(s):			No score
39		Has a senior laboratory staff member with clinical microbiology qualification been working in the lab for ≥3 last years?	Yes ☐	No ☐	Don't know ☐	5 points / 0 points / Remove
40	Bench Staff	Total number of bench staff<br><br>*(staff in microbiology/bacteriology)*	#:		Don't know ☐	No score
41		Is hands-on training of bench staff part of the routine operations for the lab?<br><br>*"hands-on" can be defined as active participation*<br>*"routine operations" can be defined as periodic (daily, weekly) and consistent work done in the lab*	Yes ☐	No ☐	Don't know ☐	3 points / 0 points / Remove
42		Do bench staff receive refresher training?	Yes ☐	No ☐	Don't know ☐	2 points / 0 points / Remove
43		When was the last refresher training on blood culture?	<2 years ☐	≥2 years ☐	Don't know ☐	3 points / 0 points / Remove
44		When was the last refresher training on Antimicrobial Susceptibility Testing (AST)?	<2 years ☐	≥2 years ☐	Don't know ☐	3 points / 0 points / Remove

Media (___/18)

45	Media currently used in lab	MacConkey	Yes ☐	No ☐			1 Point / 0 Points
46		Blood	Yes ☐	No ☐			1 Point / 0 Points
47		Chocolate	Yes ☐	No ☐			1 Point / 0 Points
48		Mueller Hinton	Yes ☐	No ☐			1 Point / 0 Points
49		Mueller-Hinton + Blood	Yes ☐	No ☐			1 Point / 0 Points
50		SS/XLD/DCA/CLED<br>*(check Yes if at least one is used)*	Yes ☐	No ☐			1 Point / 0 Points
51	Media plates	What is the source of the media plates?	Purchased ☐ *(Skip questions 52-57)*	Made in-house ☐ *(Please continue)*			10 Points / 0 Points
52	In-house media	Do you measure temperature of media before pouring?	Yes ☐	No ☐			1 Point / 0 Points
53		How is temperature measured?<br>Select all that apply.	Water bath ☐	Touch ☐	Don't know ☐	Other ☐ ____________	1 point / 0 points / Remove / 0 points
54		Where is the blood for media obtained from?<br>Select all that apply.	Sheep ☐	Other Animal ☐	Human ☐	Don't know ☐	5 points / 2 points / 0 points / Remove
55		Are autoclave tapes available and used?	Yes ☐	No ☐	Don't know ☐		1 points / 0 points / Remove
56		What is the depth of agar media in plates?	~2 mm ☐	~4 mm ☐	Don't know ☐		0 points / 1 points / Remove
57		What is the date on the last batch of plates made?	≤7 days ☐	> 7 days ☐	Don't know ☐		1 points / 0 points / Remove

Identification (__ / 20)

58	How are blood cultures performed?<br>Select all that apply.		Automated ☐	Manual ☐		5 points / 0 points
59			If automated, what machine AND model (number) is used?<br>________________________			No score
60	Do you follow an algorithm/guideline for pathogen identification?<br>*(e.g. If gram negative, do you conduct biochemical tests?)*		Yes ☐	No ☐	Don't know ☐	1 point / 0 points / Remove
61	Do you conduct…	Oxidase test	Yes ☐	No ☐	Don't know ☐	1 point / 0 points / Remove
62		Coagulase test	Yes ☐	No ☐	Don't know ☐	1 point / 0 points / Remove
63		Optochin Susceptibility test	Yes ☐	No ☐	Don't know ☐	1 point / 0 points / Remove
64	To what level of specificity, would you report Klebsiella positive samples?		Klebsiella spp ☐	Klebsiella pneumoniae ☐		0 points / 5 points
65	To what level of specificity, would you report Salmonella positive samples?		Salmonella spp ☐	Salmonella Typhi ☐		0 points / 5 points

Antimicrobial susceptibility testing (AST) (___ / 30)

66	AST Performance	How is Antimicrobial Susceptibility Testing (AST) performed? Select all that apply.	Automated (e.g., VITEK 2, Phoenix) ☐	Disk diffusion ☐	Agar dilution/ Broth microdilution ☐	AST is NOT performed ☐ *(skip questions 67-72)*	Don't know ☐	8 points / 3 point / 3 points / 0 points / 0 points
67	AST guidelines	Which guideline do you follow currently? Select all that apply.	Clinical & Laboratory Standards Institute (CLSI) ☐	European Committee on Antimicrobial Susceptibility Testing (EUCAST) ☐	Don't know ☐	Other ☐ ___________		1 point / 1 point / 0 points / 0 points (if both CLSI and EUCAST are selected, award only 1 point)
68		Have you been following this/these guideline(s) for the >3 years?	Yes ☐	No ☐	Don't know ☐			3 points / 0 points / Remove
69		Are Mueller Hinton plates used for Antimicrobial Susceptibility Testing (AST)?	Yes ☐	No ☐	Don't know ☐			1 point / 0 points / Remove
70		Are Blood-Mueller Hinton plates used for Antimicrobial Susceptibility Testing (AST) of pneumococcus?	Yes ☐	No ☐	Don't know ☐			1 point / 0 points / Remove
71		Is 0.5 McFarland cell suspension made for Antimicrobial Susceptibility Testing (AST)?	Yes ☐	No ☐	Don't know ☐			1 point / 0 points / Remove
72		When was the last time that breakpoints were updated?	<1 year ago ☐	≥1 year ☐	Don't know ☐			1 point / 0 points / Remove

73	For how many hours are fresh/new media and plates left at 37°C for, to check for contaminant growth?	48+ hours ☐		<48 hours ☐	Don’t know ☐	3 points / 0 points / Remove
74	Have you been doing this for the last 3 years?	Yes ☐		No ☐	Don’t know ☐	1 point / 0 points / Remove
75	Do you use control/standard strains, such as those from the American Type Culture Collection (ATCC) or National Collection of Type Culture (NCTC), for Internal Quality Control of Antimicrobial Susceptibility Testing (AST)?	Yes ☐		No ☐ *(skip questions 76)*	Don’t know ☐ *(skip questions 76)*	1 point / 0 points / Remove
76	If yes, at what temperature are they stored?	−20 °C ☐	−80 °C ☐	Don’t know ☐	Other ☐ ________	1 point / 0 points / Remove / 0 points
77	Have you had control/standard strains (e.g., ATCC/ NCTC) for the last 3 years?	Yes ☐		No ☐	Don’t know ☐	1 point / 0 points / Remove
78	Do you test every new batch of MacConkey agar with control/standard strains (e.g., ATCC/ NCTC) to ensure it is selective for Gram-negative growth?	Yes ☐		No ☐	Don’t know ☐	1 point / 0 points / Remove
79	Have you been testing each new batch of MacConkey agar with control/standard strains (e.g., ATCC/ NCTC) for the last 3 years?	Yes ☐		No ☐	Don’t know ☐	1 point / 0 points / Remove
80	Are control/standard strains (e.g., ATCC/ NCTC) used to test newly purchased antibiotic discs and e-strips?	Yes ☐		No ☐	Don’t know ☐	1 point / 0 points / Remove
81	If there is a problem with the quality of media, what steps are taken?	Repeat/ make/ order new batch ☐	Continue to use current batch ☐	Don’t know ☐	Other ☐ ________	5 points / 0 points / Remove / 0 points
82	Is your incubator calibrated at least one time per year?	Yes ☐		No ☐	Don’t know ☐	1 point / 0 points / Remove

Internal quality control (IQC) (___ / 38)

83	Are standard operating procedures (SOPs) in place for all laboratory procedures pertaining to sample processing for bacterial culture?	Yes ☐	No ☐		Don't know ☐	1 point / 0 points / Remove
84	Are standard operating procedures (SOPs) in place for all laboratory procedures related to pathogen identification?	Yes ☐	No ☐		Don't know ☐	1 point / 0 points / Remove
85	Are standard operating procedures (SOPs) in place for all laboratory procedures pertaining to Antimicrobial Susceptibility Testing (AST)?	Yes ☐	No ☐		Don't know ☐	1 point / 0 points / Remove
86	Do all laboratory members have access to the standard operating procedures (SOPs)?	Yes ☐	No ☐		Don't know ☐	1 point / 0 points / Remove
87	Are the standard operating procedures (SOPs) reviewed and revised periodically and been in place for at least the last 3 years?	Yes ☐	No ☐		Don't know ☐	1 point / 0 points / Remove
88	Does the lab have a contact list of vendors providing maintenance and calibration service for all instruments?	Yes ☐	No ☐		Don't know ☐	1 point / 0 points / Remove
89	Do you have back-up generator(s)?	Yes ☐	No ☐		Don't know ☐	1 point / 0 points / Remove
90	In the last three years, have any of your fridges ever malfunctioned?	Yes ☐	No ☐ *(skip questions 91)*		Don't know ☐ *(skip questions 91)*	0 point / 3 points / Remove
91	If yes, how long did it take to fix it?	1-7 days ☐	8-30 days ☐	30+ days ☐	Don't know ☐	3 points / 1 point / 0 points / Remove
92	In the last three years, did you ever NOT have access to an incubator?	Yes ☐	No ☐ *(skip questions 93)*		Don't know ☐ *(skip questions 93)*	0 point / 3 points / Remove
93	If yes, how long did it take to fix it?	1-7 days ☐	8-30 days ☐	30+ days ☐	Don't know ☐	3 points / 1 point / 0 points / Remove

External quality assurance (EQA) (___ / 78)

EQA for microbiology *is here defined as a system for objectively checking the laboratory's performance of 1) bacterial isolate/pathogen identification 2) and/or antimicrobial susceptibility testing (AMR) using an external agency or facility (national or international reference laboratory).*
Please note EQA for other lab tests (e.g. biochemistry etc.) are ***NOT*** *what we are asking here.*

94	Does the lab take part in external quality assurance (EQA) exercises for microbiology?	Yes ☐ *(Please continue)*	No ☐ *(Proceed to Visual Inspection)*	Don't know ☐ *(Proceed to Visual Inspection)*	10 points / 0 points / Remove

95	What type of External Quality Assurance (EQA)?	Proficiency test ☐	Other ☐ ________________		No score
96	Does the laboratory's method of Pathogen Identification (ID)receive EQA evaluation?	Yes ☐ *(Please continue)*	No ☐ *(Proceed to Question 106)*	Don't know ☐ *(Proceed to Question 106)*	10 points / 0 points / Remove

If lab is participating in ID EQA (if answered Yes in Question 96)						
97	Is, the EQA for Pathogen Identification (ID) provided by a national or international provider?	International ☐	National ☐	Both ☐	Don't know ☐	5 points / 3 point / 5 points / Remove
98	What is the name AND provider of the assessment(s)?	INTERNATIONAL:<br>I. Name & (Provider) ________________<br>II. Name & (Provider) ________________<br><br>NATIONAL:<br>I. Name & (Provider) ________________<br>II. Name & (Provider) ________________				No score
99	Which pathogens are included in the test panel for ID EQA? Select all that apply.	☐ Enterococcus spp.<br>☐ Staphylococcus spp.<br>☐Escherichia spp.<br>☐ Salmonella spp.<br>☐ Campylobacter spp.<br>☐ Acinetobacter spp.<br>☐ Klebsiella spp.				No score

		☐ Streptococcus spp.<br>☐ Shigella spp.<br>☐Neisseria spp.<br>☐ Other, please specify ______				
100	How frequently does the lab take part in the assessment?	Annually ☐	Bi-annually ☐	Other ☐ ______		5 points / 3 point / 0 points
101	How long has the lab taken part in the assessment?	One year or less (≤1) ☐	Between 1 to 5 years (1<5) ☐	Five years or more (≥5) ☐	Don't know ☐	1 point / 5 points / 7 points / Remove
102	Are you able to share the results of the 3 latest EQA exercises?	Yes ☐	Unable to share/confidential ☐	Don't know ☐		1 point / 0 points / Remove
103	Was any follow up exercise included?	Yes ☐	No ☐	Don't know ☐		1 point / 0 points / Remove
104	If yes, please provide details.<br>Please continue if you chose No or Don't know.					No score
105	Has your laboratory received a national or international accreditation which includes Pathogen Identification (ID)?	Yes ☐	No ☐	Don't know ☐		5 points / 0 points / Remove

106	Does the laboratory's method of Antimicrobial Susceptibility Testing (AST) receive EQA evaluation?	Yes ☐ *(Please continue)*	No ☐ *(Proceed Visual Inspection)*	Don't know ☐ *(Proceed Visual Inspection)*	10 points / 0 points / Remove

If lab is participating in AST EQA (if answered Yes in Question 106)						
107	Is, the EQA for Antimicrobial Susceptibility Testing (AST) provided by a national or international provider?	International ☐	National ☐	Both ☐	Don't know ☐	5 points / 3 point / 5 points / Remove
108	What is the name AND provider of the assessment(s)?	INTERNATIONAL:<br>I. Name & (Provider) ______<br>II. Name & (Provider) ______<br><br>NATIONAL:				No score

		I. Name & (Provider) ________________<br>II. Name & (Provider) ________________	
109	Which pathogens are included in the test panel for AST EQA?<br>Select all that apply.	☐ Enterococcus spp.<br>☐ Staphylococcus spp.<br>☐Escherichia spp.<br>☐ Salmonella spp.<br>☐ Campylobacter spp.<br>☐ Acinetobacter spp.<br>☐ Klebsiella spp.<br>☐ Streptococcus spp.<br>☐ Shigella spp.<br>☐Neisseria spp.<br>☐ Other, please specify<br>________________	No score
110	How frequently does the lab take part in the assessment?	Annually ☐ / Bi-annually ☐ / Other ☐ ________	5 points / 3 point / 0 points
111	How long has the lab taken part in the assessment?	One year or less (≤1) ☐ / Between 1 to 5 years (1<5) ☐ / Five years or more (≥5) ☐ / Don't know ☐	1 point / 5 points / 7 points / Remove
112	Are you able to share the results of the 3 latest EQA exercises?	Yes ☐ / Unable to share/confidential ☐ / Don't know ☐	1 point / 0 points / Remove
113	Was any follow up exercise included?	Yes ☐ / No ☐ / Don't know ☐	1 point / 0 points / Remove
114	If yes, please provide details.<br>Please continue if you chose No or Don't know.		No score
115	Has your laboratory received a national or international accreditation which includes Antimicrobial Susceptibility Testing (AST)?	Yes ☐ / No ☐ / Don't know ☐	5 points / 0 points / Remove

Visual Inspection

Media

Ask respondent to show you where media plates are stored.				
116	Do plates have manufacture dates clearly marked? *(Check 3 plates)*	Yes ☐	No ☐	1 point / 0 points
117	What temperature are plates stored?	4° C ☐	Other ☐ ________	1 point / 0 points

Identification

Ask respondent to show you where pathogen identification algorithm is located.				
118	Is there a pathogen identification algorithm posted in the lab? *(clearly visible above working area)*	Visible ☐	Not visible ☐	1 point / 0 points

Internal quality control

Ask respondent to show you where temperature charts are located and conduct visual observation of whether charts are kept daily for:				
119	Refrigerator(s)	Yes ☐	No ☐	1 point / 0 points
120	Freezer	Yes ☐	No ☐	1 point / 0 points
121	Incubator	Yes ☐	No ☐	1 point / 0 points

Antimicrobial susceptibility testing (AST)

These questions are regarding the Antimicrobial Susceptibility Testing (AST) guidelines currently used and how data are recorded.<br><br>***Please conduct visual observation for breakpoints and data capture.***							
122	If Clinical & Laboratory Standards Institute (CLSI) is used	For *Salmonella* Typhi, what breakpoint is used for Azithromycin?	S: ≥18 mm;<br>I: 14 - 17 mm;<br>R: ≤13 mm<br>☐	S: ≥13 mm;<br>I: n/a;<br>R: ≤12 mm<br>☐	Don’t know/ CLSI not used<br>☐		2 points / 0 points / Remove
123		For *Salmonella* Typhi, what breakpoint is used for Ciprofloxacin?	S: ≥21 mm;<br>I: 16 - 20 mm;<br>R: ≤15 mm<br>☐	S: ≥31 mm;<br>I: 21 - 30 mm;<br>R: ≤20 mm<br>☐	Don’t know/ CLSI not used<br>☐		2 points / 0 points / Remove
124		For Pneumococcus from non-meningitis cases, what breakpoint is used for Penicillin?	S: ≤0.06 ug/ml;<br>I: 0.12-1 ug/ml;<br>R: ≥2 ug/ml<br>☐	S: ≤2 ug/ml;<br>I: 4 ug/ml;<br>R: ≥8 ug/ml<br>☐	Don’t know/ CLSI not used<br>☐	MIC is not performed<br>☐	2 points / 0 points / Remove / Remove
125	Data capture	Are the data stored as:<br>Sensitive (S),<br>Intermediate (I),<br>Resistant (R)	Yes<br>☐	No<br>☐	Don’t know<br>☐		5 points / 0 points / Remove
126		Are exact values of zone diameters or minimum inhibitory concentration (MIC) stored?	Yes<br>☐	No<br>☐	Don’t know<br>☐	MIC is not performed<br>☐	3 points / 0 points / Remove
