# Supplementary material for: Methodological Approach to Identify and Expand the Volume of Antimicrobial Resistance (AMR) Data in the Human Health Sector in Low- and Middle-Income Countries in Asia: Implications for Local and Regional AMR Surveillance Systems Strengthening
Source: Clin Infect Dis. 2023 Dec 20;77(Suppl 7):S507–18. doi: 10.1093/cid/ciad634 (PMC10732564; doi:10.1093/cid/ciad634)
Supplement: ciad634_Supplementary_Data [file ciad634_supplementary_data.zip › Appendix 6. CAPTURA AMR readme file template.pdf]

Section	Items	Responses
Details on file:	File name	
	CAPTURA ID	[IVI to fill out this section]
	Facility Name	
	Type of data [AMR/U/C/c]	AMR
	Name of person who uploaded dataset to Warehouse<br><br>Date of upload (dd/mm/yyyy)	
	Name of person completing this template	
	Country	
	OTHER REMARKS	
Data description:	Number of data variables (columns)	
	Number of observations (rows)	
	Is a data dictionary available (Yes /No/ Don't know)?	
	Is the dictionary uploaded in the warehouse (Yes /No/ Don't know)?	
	Is there any other associated files/documentation uploaded in the warehouse (Yes /No/ Don't know)?<br><br>If yes, please give brief description.	
	Time-period of dataset (e.g., month and year range)<br><br>If the dataset is made of multiple years please specify the period for each year (e.g., 2016 – Jan to July,<br><br>2017 – Jan to Sept)	
	Geographic area of dataset<br><br>Please specify as much as possible (e.g., wards of hospital, district/town facility generally serves)	

	OTHER REMARKS	

AMR data<br>*Please note these questions are asking for information during the original time of data collection (2016-19)*<br>*Please distinguish "No/None" from "Unknown/Don't know""*		
Data quality	Are there any data duplicated across files?	
	Is the data complete (e.g., are all data included or just the first 10 per month)?	
	Are negative testing results included (e.g., no pathogen detected)?	
	Are number of non-viable or destroyed samples available (e.g., samples which couldn't be cultured or identified)?<br>If yes, please describe as best to your knowledge.	
	What criteria were used to collect data (e.g., only blood samples, only in-patient)?	
	Were there any significant changes which may have affected the data available in each file?<br>E.g.,<br>- New equipment introduced (e.g., Vitek)<br>- Quality control system introduced/enhanced<br>- Changes in guidelines used (e.g., CLSI to EUCAST)<br>- Changes in testing protocols (e.g., not testing urine)	
	Was there any testing for new antimicrobials (panels added/expanded)?	

	Was testing interrupted or changed during the data collection period<br><br>(e.g. machine X was broken in June 2018 so we couldn't test Y)?	
	OTHER REMARKS	
	Please describe how you gathered the information provided above	
Denominators	Population data for area	
		Sources of denominator:
Approximate values are accepted	Number of hospital/ward in-patient days (per day/month/year)	
		Sources of denominator:
	Number of yearly admissions (per year)	
		Sources of denominator:
	Number of hospital out-patient visits (per day/month/year)	
		Sources of denominator:
	Number of bacterial cultures processed (per day/month/year)	
		Sources of denominator:
	Number of blood cultures processed (per day/month/year)	
		Sources of denominator
	[If you can get other similar and useful information not listed here please include here]	
		Sources of denominator:
CAPTURA data quality indicators	*RLQA score*	[IVI to fill out this section]
