# Supplementary material for: Methodological Approach to Identify and Expand the Volume of Antimicrobial Resistance (AMR) Data in the Human Health Sector in Low- and Middle-Income Countries in Asia: Implications for Local and Regional AMR Surveillance Systems Strengthening
Source: Clin Infect Dis. 2023 Dec 20;77(Suppl 7):S507–18. doi: 10.1093/cid/ciad634 (PMC10732564; doi:10.1093/cid/ciad634)
Supplement: ciad634_Supplementary_Data [file ciad634_supplementary_data.zip › Appendix 8. CAPTURA AMC readme file template.pdf]

Section	Items	Responses
Details on file:	File name	
	CAPTURA ID	[IVI to fill out this section]
	Facility Name	
	Type of data [AMR/U/C/c]<br><br>Please indicate AMC Macro or micro	
	Name of person who uploaded dataset to Warehouse<br><br>Date of upload (dd/mm/yyyy)	
	Name of person completing this template	
	Country	
	OTHER REMARKS	
Data description:	Number of data variables (columns)	
	Number of observations (rows)	
	Is a data dictionary available (Yes /No/ Don't know)?	
	Is the dictionary uploaded in the warehouse (Yes /No/ Don't know)?	
	Is there any other associated files/documentation uploaded in the warehouse (Yes /No/ Don't know)?<br><br>If yes, please give brief description.	
	Time-period of dataset (e.g., month and year range):<br><br>If the dataset is made of multiple years please specify the period for each year (e.g., 2016 – Jan to July,<br><br>2017 – Jan to Sept)	
	Geographic area of dataset<br><br>Please specify as much as possible (e.g., wards of hospital, district/town facility generally serves)	

	OTHER REMARKS	

AMC/c data<br>*Please note these questions are asking for information during the original time of data collection (2016-19)*<br>*Please distinguish "No/None" from "Unknown/Don't know""*		
Data quality	Are there any data duplicated across files?	
	Is the data complete (e.g., are all data included or just the first 10 per month)?	
	What other criteria are used?<br>E.g.,<br>- data from only IPD/OPD/surgical wards<br>- use of standard template from govt/facility	
	Were there any significant changes which may have affected the data available in each file?<br>E.g.,<br>- Changes in data entry (e.g., use of software)<br>- Changes in protocols (e.g., updates in govt/facility guidelines)?	
	OTHER REMARKS	
	Please describe how you gathered the information provided above	
Macro AMC Denominators<br>*Approximate values are accepted*	Country population data	 Sources of denominator:
	[If you can get other similar and useful information not listed here please include here]	 Sources of denominator:

Micro AMC Denominators	Population data at distribution level, if available (e.g., district)	Sources of denominator:
Approximate values are accepted	[If you can get other similar and useful information not listed here please include here]	
		Sources of denominator:
CAPTURA data quality indicators	*RDQA score*	[IVI to fill out this section]
