# Supplementary figures and images for: Methodological Approach to Identify and Expand the Volume of Antimicrobial Resistance (AMR) Data in the Human Health Sector in Low- and Middle-Income Countries in Asia: Implications for Local and Regional AMR Surveillance Systems Strengthening
Source: Clin Infect Dis. 2023 Dec 20;77(Suppl 7):S507–18. doi: 10.1093/cid/ciad634 (PMC10732564; doi:10.1093/cid/ciad634)

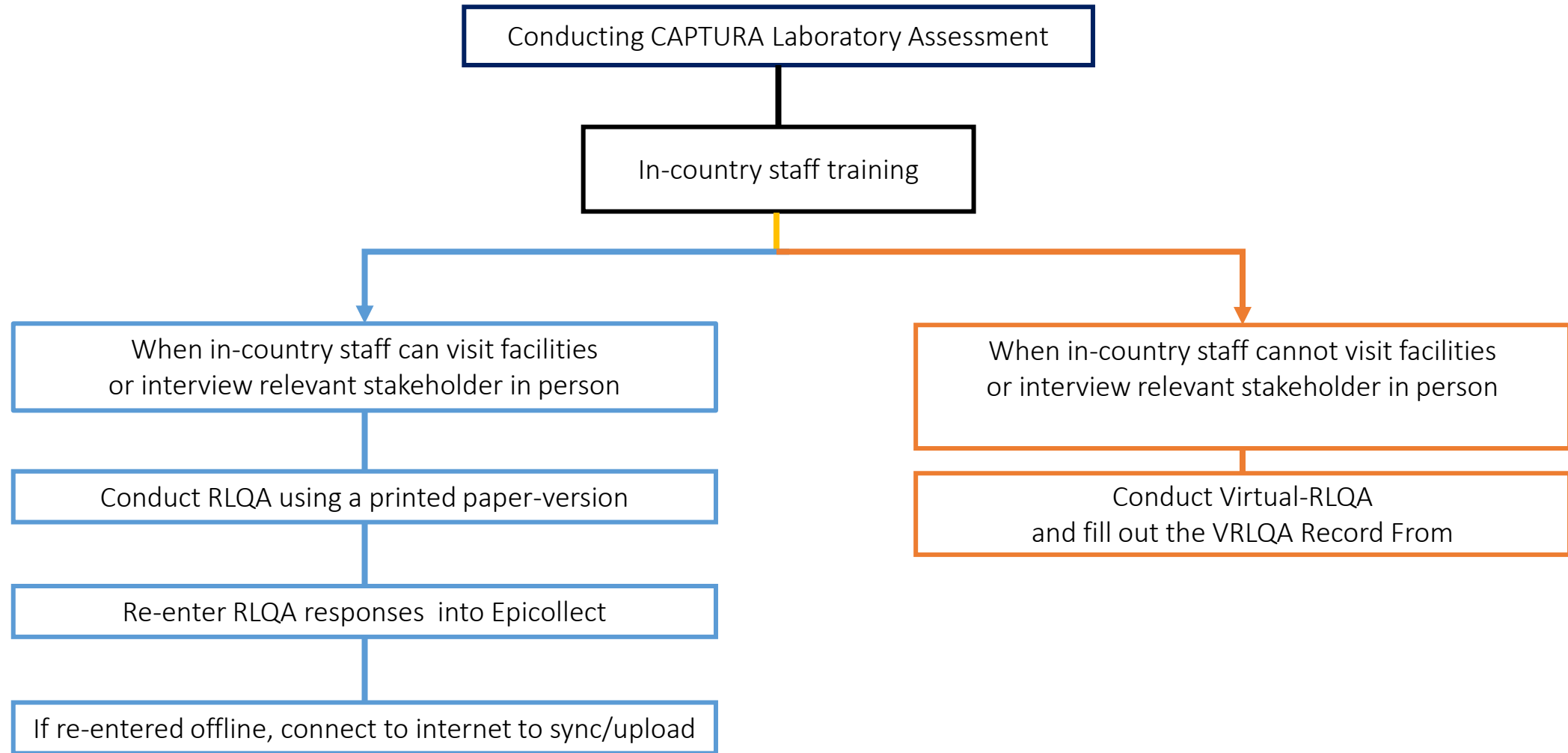

Supplement: ciad634_Supplementary_Data [file ciad634_supplementary_data.zip › Appendix 5. CAPTURA RLQA deployment method.pdf]
